# CaaX-less lamins: Lophotrochozoa provide a glance at the playground of evolution

**DOI:** 10.1007/s00709-022-01809-3

**Published:** 2022-09-14

**Authors:** Reimer Stick, Annette Peter

**Affiliations:** Department of Cell Biology, University of Bremen, P.O. Box 330440, 28334 Bremen, Germany

**Keywords:** Lamins, CaaX motif, CaaX-less lamins, Lophotrochozoa, Alternative C-termini, Membrane association

## Abstract

Nuclear lamins are the main components of the nuclear lamina in many eukaryotes. They are members of the intermediate filament (IF) protein family. Lamins differ from cytoplasmic IF proteins by the presence of a nuclear localisation sequence (NLS) and a C-terminal tetrapeptide, the CaaX motif. The CaaX motif is target of post-translational modifications including isoprenylation, proteolytic processing, and carboxyl-methylation. These modifications, in conjunction with the NLS, direct lamins to the inner nuclear membrane where they assemble into filaments. Lamins lacking a CaaX motif are unable to associate independently with nuclear membranes and remain in the nucleoplasm. So far, three species have been reported to exclusively express CaaX-less lamins. All three belong to the lophotrochozoan lineage. To find out whether they represent rare exceptions, we analysed lamins of representatives of 17 lophotrochozoan phyla. Here we report that all four clades of Rotifera as well as individual taxa of Mollusca and Annelida lack CaaX-lamins, but express lamins with alternative C-termini. Of note, the respective mollusc and annelid groups occupy very different phylogenetic ranks. Most of these alternative C-termini are rich in aromatic residues. A possible function of these residues in membrane association is discussed. Alternative splicing of terebellid lamin transcripts gives rise to two lamin variants, one with a CaaX motif and one with an alternative C-terminus. A similar situation is found in Arenicolidae, Opheliidae, Capitellidae, and Echiura. This points a way, how the switch from lamins carrying a CaaX motif to lamins with alternative C-termini may have occurred.

**Supplementary Information:**

The online version contains supplementary material available at 10.1007/s00709-022-01809-3.

## Introduction

The nuclear lamina is an integral part of the nuclear skeleton in eukaryotic cells. The lamina is made up of a network of filaments that are closely attached to the nuclear aspect of the inner nuclear membrane. Lamin proteins constitute the major components of the lamina. Together with lamina-associated polypeptides and lamin-binding proteins, they contribute to a wealth of cellular functions. The lamina is involved in nuclear mechanics and nuclear positioning, in the spatial organization of interphase chromatin and DNA replication, and it contributes to transcription and regulatory functions (Burke and Stewart 2013; Gruenbaum and Foisner 2015; Patil and Sengupta 2021). Mutations in lamin genes and their associated proteins cause a wide range of severe human hereditary diseases underscoring their relevance for the integrity of cellular functioning (Prokocimer et al. 2009; Wong and Stewart 2020).

Lamin proteins are members of the intermediate filament (IF) protein family (Peter et al. 1990). Most non-vertebrate genomes contain only a single lamin gene; exceptions are mosquitoes and fruit flies, which harbour two lamin genes. Vertebrates, on the other hand, possess three (mammals) or four (all other vertebrates) lamin genes (Peter and Stick 2012; Stick and Peter 2020).

All IF proteins are characterized by a tripartite domain organization. A short head domain and a larger tail domain flank a central α-helical domain, the rod domain. The rod domain forms an α-helical coiled-coil. In metazoans, it is subdivided into three α-helical subdomains, each of characteristic length and separated by short non-helical linkers (Herrmann and Aebi 2016). Conserved sequence motifs at both ends of the rod domain trigger coiled-coil formation and assembly into higher order filaments (Chernyatina et al. 2015; de Leeuw et al. 2018). The tail domains of lamins and of non-vertebrate cytoplasmic IFs contain a highly distinctive immunoglobulin-like fold, the lamin tail domain (LTD) (Dhe-Paganon et al. 2002). Phosphorylation/dephosphorylation at a cyclin-dependent kinase 1 recognition site within the lamin head domain causes disassembly and reassembly of the lamina during mitosis, respectively.

Lamins can be distinguished from cytoplasmic IF proteins by the presence of a nuclear localisation sequence (NLS) and by a CaaX isoprenylation motif at the C-terminus (Peter and Stick 2015). The CaaX tetrapeptide with the consensus (cysteine-aliphatic-aliphatic-methionine) is post-translationally isoprenylated at its cysteine residue, followed by endoproteolytic removal of the aaX residues, and subsequent carboxyl-methylation of the now terminal cysteine residue (Nigg et al. 1992). These modifications in conjunction with the NLS target lamins to the inner nuclear membrane where they form filaments (Loewinger and Mckeon 1988; Holtz et al. 1989). Ablation of the CaaX motif or mutation of the CaaX cysteine residue disables membrane association (McKeon 1991).

A few organisms express lamin proteins that lack a CaaX motif. Examples for CaaX-less lamins are the mammalian C lamins (Fisher et al. 1986), the C lamins of the Brachycera, a suborder of the dipterans (Bossie and Sanders 1993; Kollmar 2015), and the barley midge *Mayetiola destructor*, a dipteran belonging to the order Nematocera (Table 1) (Brandt 2015). Notably, in all these cases the CaaX-less lamin is co-expressed with at least one CaaX-containing lamin protein. This is interpreted in the way that CaaX-less lamins reach the nuclear membrane by co-transport with CaaX-lamins. Experimental settings in which co-expression of a CaaX-lamin is inhibited, demonstrate that CaaX-less proteins on their own are not able to associate with membranes, but remain in the nucleoplasm (Krohne et al. 1989). However, other mechanisms of membrane association are known for CaaX-less lamins, like the C2 lamins of mammals. C2 lamins are selectively expressed in male germ cells and acquire membrane affinity by myristoylation of a glycine residue at their N-terminus (Alsheimer et al. 2000).

**Table 1:** Re-evaluation of the presence of a C-terminal CaaX motif in lamin proteins

Phylum	Class	Species	NCBI:txid	Number of lamins	Gene	length aa	ORF complete	NCBI accession	NLS	LTD	C-terminal sequence	Type of database
	Choano-flagellata	*Salpingoeca rosetta*	txid946362	2	Yes	556	Yes	NW_004754902/PTSG_10006/SRR100060.2158821.3/.1512940.3/.1800333.3	Yes	Yes	ESSTWAIMRD*	WGS/RNA-Seq
					Yes	540	Yes	NW_004754902/PTSG_10008/ACSY01003084/SRR100054.266225.3/SRR100055.1019350.3/.1142367.3/.1736989.3/SRR100062.187582.3/SRR100066.53865.3	Yes	Yes	CSLM*	WGS/RNA-Seq
Porifera	Demospongiae	*Amphimedon queenslandica*	txid400682	1	Yes	607	Yes	ACUQ01005773.1/XM_011407353.2	Yes	Yes	CIIS*	WGS
Arthropoda	Hexanauplia	*Eurytemora affinis*	txid88015	1	Yes	545	Yes	AZAI01121165.1/GEAN01055336.1	Yes	Yes	CVIM*	WGS/TSA
Arthropoda	Hexanauplia	*Lepeophtheirus salmonis*	txid72036	1	Incomplete	595	Yes	HACA01007752.1	Yes	Yes	CSVM*	TSA

Abbreviations: *aa*, amino acid residues; *NLS*, monopartite nuclear localisation signal; *LTD*, lamin tail domain; *WGS*, whole genome shotgun contigs; *TSA*, transcriptome shotgun assembly; *RNA-Seq*, RNA sequencing using next-generation sequencing; *the C-terminus of a polypeptide

Sequence analysis of an increasing number of phylogenetically very different organisms has shown that lamin proteins, originally characterized in metazoans also occur in other branches of the eukaryotic tree. Although sequence information is still incomplete, especially for unicellular eukaryotic taxa, it is assumed that a lamin gene was already present in the last eukaryotic common ancestor (LECA) (Krüger et al. 2012; Kollmar 2015; Koreny and Field 2016). The search for lamins has also revealed that lamin proteins are missing in certain eukaryotic branches where their function is taken over by other proteins. Examples are the NMCP proteins in plants (Streptophyta), e.g. NMPCs of *Daucus carota* (Masuda et al. 1997; Ciska and de la Espina 2013), CROWDED NUCLEI (CRWN 1–4) of *Arabidopsis thaliana* (Wang et al. 2013), and the NUP-1 protein in trypanosomes, which are the main components of the lamina in these organisms (Rout and Field 2001). NMCPs and NUP-1 have analogous functions, but do not show phylogenetic relationship to lamins of the IF protein family (Koreny and Field 2016).

Extensive search for lamin sequences has unexpectedly revealed the existence of organisms that only possess CaaX-less lamins. Examples are the annelids *Capitella teleta* (Zimek and Weber 2011) and *Helobdella robusta* (Kollmar 2015) as well as the rotifer *Adineta vaga* (Kollmar 2015). All three species are members of the monophyletic group of Lochotrophozoans. The same has been reported for the choanoflagellate *Salpingoeca rosetta*, the sponge *Amphimedon queenslandica*, and one of the lamins of the rotifer *Adineta vaga* (Kollmar 2015). The lamins of *Amphimedon queenslandica* and one of the lamins of the rotifer *Adineta vaga* have been reported to additionally lack the LTD. (Kollmar 2015). An even more extensive loss of lamin sequence motifs was reported for species of two different crustacean orders, *Lepeophtheirus salmonis* and *Eurytemora affinis*. The latter were described to contain a conserved protein interaction domain, a PDZ domain, in their N-termini and to lack the entire C-terminus, which carries the LTD and the CaaX motif in other lamins (Kollmar 2015). None of these proteins carries other readily apparent recognition sites for lipidation, such as an N-terminal palmitoylation site. It therefore remains open how these proteins might associate with the inner nuclear membrane. To clarify whether these cases represent special biological cases or are merely due to missing sequence information or incorrect annotations we re-analysed these sequences and extended our search for CaaX-less lamins to more representatives of the lochotrophozoan lineage.

## Materials and methods

Lamin sequences were retrieved from the NCBI databases (Agarwala et al. 2016) and the MolluscaBase (MolluscaBase 2020) by blast searches, using protein sequences of various lamins as query. Lophotrochozoan transcriptomes and proteomes were kindly provided by authors of the following publications: (Zverkov et al. 2019), (Ogura et al. 2013; Halanych and Kocot 2014; Struck et al. 2014; Lu et al. 2017; Laumer et al. 2019; Marlétaz et al. 2019; Stiller et al. 2020; Tilic et al. 2020, 2022; Mauer et al. 2021) and were searched by tblastn and blastp using lamin sequences of species from closely related taxa. Sequence alignments were done using MultAlin software (Corpet 1988). Protein statistics were done with The Sequence Manipulation Suite (Stothard 2000).

## Results

### Re-analysis of lamin sequences missing a CaaX motif

The loss of lamin-specific sequence motifs in only a single species within a systematic group, whose other members carry these motifs, raises questions of its biological significance. We therefore re-analysed the lamin sequences of the respective species, using information from publicly accessible databases. In some cases, we carried out de novo transcriptome assemblies using published RNA-Seq data.

For the choanoflagellate *Salpingoeca rosetta*, we have obtained the following: the genes encoding the two Salpingoeca lamins, lamin-1 and lamin-2, are arranged in tandem within the same genomic region. The upstream gene encodes the CaaX-less lamin-1 (PTSG_10006); the downstream gene contains a CaaX-encoding lamin (PTSG_10008); however, the last exon encoding the CaaX motif is located on a separate genomic contig (ACSY01003084) and therefore probably remained undiscovered so far (Table 1). The assignment of this exon to the *S. rosetta* lamin-2 gene is corroborated by transcript mapping, which shows that all exon/exon junctions, including that of the CaaX-encoding exon, are covered by RNA-Seq reads (Table 1). The CaaX-less lamin is transcribed, as shown by RNA-Seq data. In addition, we identified lamin transcripts for a further 10 choanoflagellate species by searching the iMicrobe database (Youens-Clark et al. 2019). All 10 species encode lamins carrying a CaaX motif. Taken together, this suggests that a gene duplication of the lamin gene has taken place in *Salpigoeca rosetta* and that one of the two lamins genes has lost the CaaX motif.

For the sponge *Amphimedon queenslandica*, we published previously a complete lamin sequence containing both an LTD and a CaaX motif (Peter and Stick 2012) (Table 1). The identical sequence is listed in the NCBI database as predicted Amphimedon lamin-L(I)-like, supported by 100% coverage of the annotated genomic features by RNA-Seq alignments (XM_011407353.2).

For *Lepeophtheirus salmonis* and *Eurytemora affinis*, transcripts encoding *bona fide* lamin proteins with an NLS, an LTD, and a CaaX motif were retrieved from the NCBI transcriptome database (Table 1). Sequences similar to those described by Kollmar (Kollmar 2015), which carry a PZD domain and coiled-coil domains but lack any further lamin features, can be found in many other crustaceans, but these genes do not belong to the lamin gene family.

### CaaX-less lamins in the lophotrochozoan lineage

Three species remain, which have been reported to have lamin proteins, of which none carries a CaaX motif, *Capitella teleta*, *Helobdella robusta*, and *Adineta vaga*. All three belong to the clade Lophotrochozoa (Halanych et al. 1995; Kocot 2016; Marlétaz et al. 2019). Two Capitella lamin variants will be described in the section ‘CaaX-less lamins in the phylum Annelida’. *Helobdella robusta* expresses two lamins, both lack a CaaX motif, as consistently proven by transcriptomic and genomic sequences (Table 4). The lack of CaaX motifs in *Adineta vaga* lamins is reported in the section ‘Rotifera syn. Syndermata’.

Since the absence of a CaaX motif in the lamin proteins of two species could be proven beyond doubt, and since these species belong to the lophotrochozoan clade we next searched for lamin sequences in closely related species and then extended our search to the entire group of Lophotrochozoa.

**Fig. 1:**
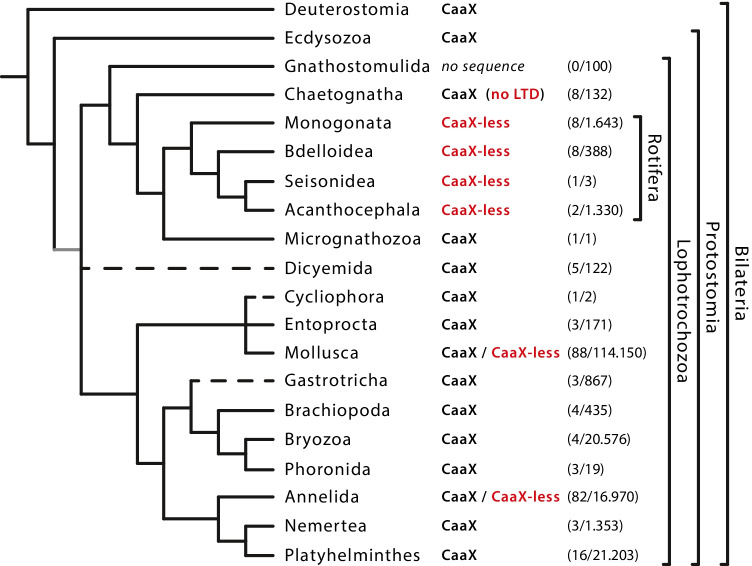
Phylogenetic relationships of the lophotrochozoan superclade modified after Bleidorn (2019). CaaX or CaaX-less denotes the presence or absence of the C-terminal CaaX motif in lamin proteins of the respective phyla. In molluscs and annelids, this motif is only absent in individual taxa (CaaX/CaaX-less). Lamins of the Chaetognatha lack a lamin tail domain (no LTD). For Gnathostomulida, sufficient sequence data are still missing (*no sequence*). The number of species for which lamin sequences were obtained is given in parentheses together with the total number of species of this taxon as listed in the Catalogue of Life (Bánki et al. 2022). The two other bilaterian groups, the Ecdysozoa, the sister group of the Lophotrochozoa, and the Deuterostomia are also indicated

Lophotrochozoa is one of the three major monophyletic groups of bilaterian animals (Halanych et al. 1995). The other two are the Ecdysozoa and the Deuterostomia. The Ecdysozoa are the sister group of the Lophotrochozoa. Together they constitute the monophyletic group Protostomia. (Fig. 1) (Halanych et al. 1995; Kocot 2016; Bleidorn 2019; Marlétaz et al. 2019). Ecdysozoa include the nematodes and arthropods, while the Deuterostomia include the hemichordates, echinoderms, and chordates. The clade Lophotrochozoa is home to 18 phyla with taxa as diverse as the segmented annelids, the shell-bearing molluscs and brachiopods, the colony-forming bryozoans and the endoparasitic Acanthocephala, to name but a few (Halanych et al. 1995; Kocot 2016). They include both species-rich groups and groups consisting of only a few species (Bánki et al. 2022).

We were able to determine lamin sequences of representatives from 17 of 18 lophotrochozoan phyla (Fig. 1), totalling more than 200 species (Supplementary Table S1). Our analysis reveals that CaaX-less lamins occur in at least six clades of different phylogenetic rank, namely within the Annelida, the Mollusca, and all four clades of the Rotifera (Fig. 1).

### Rotifera syn. Syndermata

The phylum Rotifera includes the Monogonata, Bdelloidea, Seisonidea, and Acanthocephala (Fig. 1) (García-Varela and Nadler 2006; Struck et al. 2014; Wey-Fabrizius et al. 2014; Sielaff et al. 2016). Members of these four groups have very different lifestyles and different modes of reproduction. Most monogononts and bdelloids are free-living aquatic organisms. Monogononts have a heterogontic life cycle switching between parthenogenetic and bisexual reproduction (Gilbert 2020), while bdelloids reproduce exclusively parthenogenetically. They lack males and there exists no evidence of meiotic processes (Fontaneto et al. 2007). Acanthocephalans are obligate endoparasites with a life cycle switching between invertebrates as intermediate and vertebrates as final hosts. Seisonids, with only two species known so far, ectoparasitize on marine crustaceans of the genus Nebalia and reproduce strictly sexually (Mauer et al. 2021). Despite this great diversity, Rotifera form a monophyletic group. One of the synapomorphic characters is the presence of a syncytial epidermis, hence the synonym Syndermata.

Lamin sequences have been obtained for representatives of all four rotiferan clades, namely for eight bdelloids (Flot et al. 2013; Eyres et al. 2015), eight monogononts (Hanson et al. 2013; Lee et al. 2015; Gribble and Welch 2017; Kang et al. 2020; Kim et al. 2021), two acanthocephalans (Struck et al. 2014; Mauer et al. 2020), and for *Seison nebaliae* (Mauer et al. 2021) (Supplementary Table S1). None of these lamins carries a CaaX motif. Moreover, the C-terminal sequences differ markedly between individual rotiferan groups. The genome of bdelloid *Adineta vaga* is tetraploid; its structure is incompatible with conventional meiosis (Flot et al. 2013). The Adineta genome contains four lamin genes, reflecting its tetraploid state. Two of the four genes are almost identical to each other. Of note, all four lamins possess an LTD but all lack a CaaX motif (Table 2). We obtained transcriptomic and genomic sequence information for two additional Adineta species and five bdelloid species of other genera. C-termini of bdelloid lamins are characterized by a high proportion of aliphatic amino acid residues (40–50%), but lack aromatic residues (Table 2). C-termini of monogonont lamins, in contrast are rich in aromatic residues (phenylalanine and tryptophan) (Table 2). While the proportion of aromatic residues in total lamin proteins averages about 5%, up to four out of the last 10 residues (10–40%) are aromatic in monogonont lamins (Table 2 and Supplementary Table S1). Moreover, with the exception of the *Brachionus calyciflorus* lamin, the C-termini are devoid of negatively charged residues. *Pomphorhynchus laevis*, an acanthocephalan, and the seisonid *Seison nebaliae* each express two very similar lamin proteins. In Seison, alternative splicing gives rise to a total of four protein variants, three of which differ in their C-termini. One of these variants carries the tetrapeptide sequence CSEE at its C-terminus. Despite the presence of the cysteine residue in an appropriate position, this sequence cannot serve as an isoprenylation substrate. This is because the farnesyltransferase cannot accommodate charged residues such as the glutamic acid residue in the X-position in its specificity pocket (Reid et al. 2004).

**Table 2:** Amino acid composition of Rotifera lamin C-termini

	Species	C-terminus	aliph	arom	pos	neg	tiny
Monogonata	*Brachionus koreanus*	KFLGLWKSQP*	20	20	20	0	20
	*Brachionus angularis*	VNKFFNLWKN*	20	30	20	0	0
	*Brachionus calyciflorus*	VDKDANVSFI*	30	10	10	20	20
	*Brachionus manjavacas*	AVQKFLGLWK*	30	20	20	0	20
	*Brachionus paranguensis*	AVQKFLGLWK*	30	20	20	0	20
	*Brachionus plicatilis*	AVQKFLGLWK*	30	20	20	0	20
	*Brachionus rotundiformis*	QKFLGLWKTG*	22	22	22	0	22
	*Proales similis*	SSRKFFAFWK*	0	40	30	0	30
Acanthocephala	*Macracanthorhynchus hirudinaceus*	VVDIDFLRIL*	60	10	10	20	0
	*Pomphorhynchus laevis-1*	SRLFNLFQNN*	20	20	10	0	10
	*Pomphorhynchus laevis-2*	PMDPSEKNSI*	10	0	10	20	20
	*Seison nebaliae-1a*	VQYSMN**C**SEE*	10	10	0	20	20
	*Seison nebaliae-1b*	QKLVRSRKLN*	30	0	40	0	10
Seison	*Seison nebaliae-2*	YAMEYNTVVE*	20	20	0	20	10
	*Adineta ricciae*	VVAEKVVSVK*	50	0	20	10	20
	*Adineta vaga-1a/b*	VVAEKIVTLK*	50	0	20	10	10
Bdelloidea	*Adineta vaga-2a/b*	VVAEKIVTVK*	50	0	20	10	10
	*Didymodactylos carnosus-1*	DDDCKVDKSI*	20	0	20	40	10
	*Didymodactylos carnosus-2*	IVAEKILTIK*	50	0	20	10	10
	*Rotaria magnacalcarata*	VIVEKTVTVK*	50	0	20	10	0
	*Rotaria rotatoria*	VIAEKTVTVK*	40	0	20	10	10
	*Rotaria socialis*	VIAEKTVTVK*	40	0	20	10	10
	*Rotaria sordida*	IVAEKTITIK*	40	0	20	10	10
	*Rotaria tardigrada*	VVAEKTITIK*	40	0	20	10	10

Amino acid composition in % of the 10 C-terminal residues of lamins of the indicated species. *aliph*, aliphatic (I, L, V); *arom*, aromatic (F, W, Y); *pos*, positive (K, R, H); *neg*, negative (D, E); tiny (G, A, S) residues. Since not all 20 amino acids can be assigned to these groups, the sum does not add up to 100% in all cases. *The C-terminus of a polypeptide

### Mollusca with emphasis on Cephalopoda

**Fig. 2:**
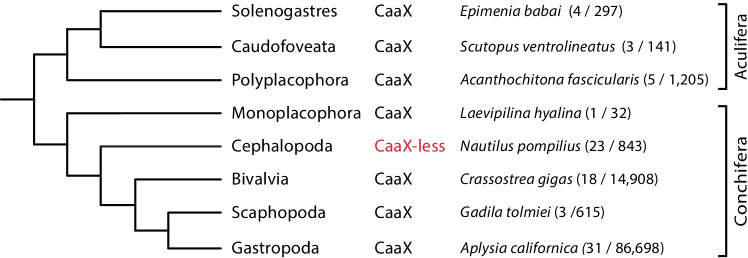
Phylogenetic relationships of the 8 classes of Mollusca, modified after Kocot et al. (2020). CaaX or CaaX-less denotes the presence or absence of the C-terminal CaaX motif in lamin proteins of the respective classes. Species name of one representative for each class is given. The number of species for which lamin sequences were obtained is given in parentheses together with the total number of species of this class as listed in the MolluscaBase (MolluscaBase 2020)

The phylum Mollusca comprises eight classes, including very species rich classes with more than 10,000 species (Gastropoda, Bivalvia, and Cephalopoda), those with several hundred species (Polyplacophora, Scaphopoda, Solenogastres, Caudofoveata), and the Monoplacophora, for which only 32 species have been described to date (MolluscaBase (2020) (Fig. 2). The phylogenetic relationships among the eight clades have been largely clarified (Wanninger and Wollesen 2019; Kocot et al. 2020) (Fig. 2). Only the position of the Monoplacophora is still subject of debate. By morphological criteria they are considered a sister group to the rest of the Conchifera (Fig. 2), while phylogenomic analyses places them together with Cephalopoda (Kocot et al. 2020). Lamin sequences were obtained for representatives of all eight classes. But the number of species for which lamin sequences have been obtained varies greatly among the eight classes (Fig. 2). This depends, among other things, on the species richness of a group as well as on the sequencing depth, which in turn usually reflects the medical, economic or scientific importance of the respective group. The latter is especially the case when a species/clade holds a unique phylogenetic position.

**Fig. 3:**
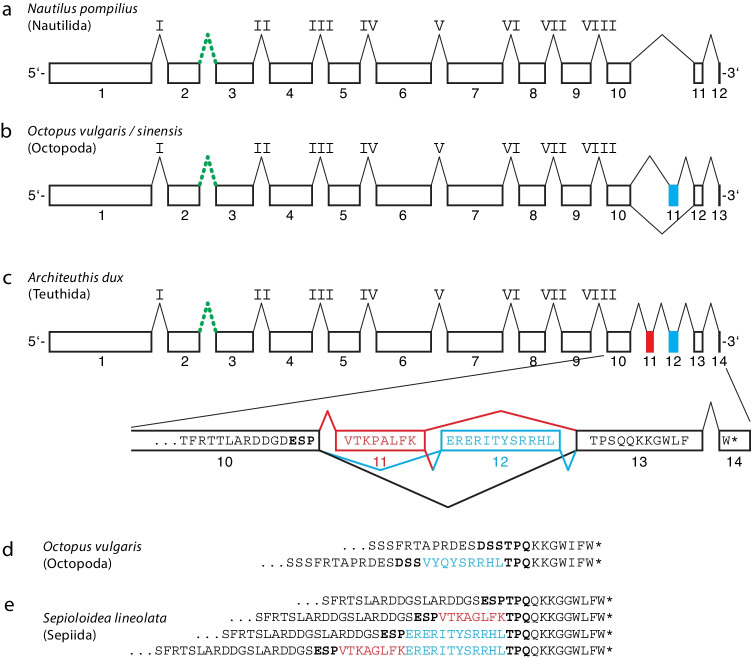
Schematic representation of the exon/intron pattern of lamin genes of three cephalopods: (a) *Nautilus pompilius* (Nautilida), (b) *Octopus vulgaris* (Octopoda), (c) *Architeuthis dux* (Teuthida). No transcript information is available for *Architeuthis dux*. However, the Architeuthis gene structure could be deduced by using the transcript information of *Sepioloida lineolata*, for which four transcript variants are known (e). Open boxes with the corresponding Arabic numerals below the boxes represent exons. Their length corresponds approximately to the length of the respective coding regions. The alternatively spliced exon 11 in (b) and 11 and 12 in (c) are drawn in red and blue, respectively. The exon encoding the last two nucleotides of the terminal tryptophan codon is represented by a vertical black bar. Intron positions are each represented by an inverted V. Archetypal lamin introns are numbered by Roman numerals I to VIII according to (Peter and Stick 2012). The second intron, represented as a green broken inverted V is exclusively found in protostomes, to which the cephalopods belong. The blow-up in (c) shows the last 15 amino acid residues encoded by exon 10 and the complete sequences encoded by exons 11 to 14 of the Architeuthis lamin gene. Asterisks (*) indicate termination codons. Of note, the highly conserved terminal tryptophan residue is encoded by two exons. The first nucleotide of the triplet is encoded by exon 13, the second and third nucleotide by exon 14; this split is not represented in the schematic for the sake of clarity. In (d) and (e) are shown the C-terminal sequences encoded by alternative spliced transcript variants of (d) *Octopus vulgaris* and (e) *Sepioloidea lineolata*, respectively. For *Sepioloidea lineolata*, only transcriptome data are currently available. However, the mode of splicing of these transcript variants can be deduced with the help of the Architeuthis lamin gene structure (c). Note that in *Sepioloidea linoleolata* all four possible splice options are realised (e)

Lamins with a CaaX motif were consistently found in all mollusc classes, with the notable exception of cephalopods (Fig. 2). In all 23 cephalopod species studied, only lamins lacking this motif were identified. These 23 species represent six orders, including Nautilus at the very base of the cephalopod phylogenetic tree (Uribe and Zardoya 2017). Of note, all cephalopod lamins possess a 10–12 amino-acid-residue-long highly conserved C-terminal sequence rich in aromatic residues with the sequence consensus: TPsQqkkg**W**lFW* (uppercase ≥ 90% identity, lowercase ≥ 50% identity) (Corpet 1988). We identified complete lamin genes for five cephalopod species for which whole genome assemblies of high quality were available, namely *Nautilus pompilius* (Ogura et al. 2013; Zhang et al. 2021), of the two closely related species of the common Octopus complex (Gleadall 2016), i.e. *Octopus sinensis* (Li et al. 2020) and *Octopus vulgaris* (Zarrella et al. 2019), the gene of *Octopus minor* (Kim et al. 2018), and of *Architeuthis dux* (da Fonseca et al. 2020). We determined the exon–intron patterns for these five genes, three of which are shown (Fig. 3a, b, c). No transcript information is available for *Architeuthis dux* (da Fonseca et al. 2020). However, the gene structure could be deduced by using the transcript information of *Sepioloida lineolata*, for which four transcript variants are known (Fig. 3e). The coding region of the Nautilus lamin gene is made up of 12 exons separated by 11 introns (Fig. 3a). Eight of these introns correspond in position to archetypal lamin introns found in many metazoan lamin genes including non-bilaterians and bilaterians (Peter and Stick 2012) (labelled with Roman numerals I to VIII in Fig. 3). In contrast, the intron that separates exon 2 from exon 3 is restricted to the Protostomia lineage (Peter and Stick 2012). This pattern was also found in the other four cephalopod lamin genes.

Downstream of exon 10, cephalopod lamin genes differ from those of most other species. While in most other metazoans the CaaX motif is encoded in a single short exon, C-termini of the cephalopod lamins are encoded by two, three or four exons respectively (Fig. 3a, b, c). The C-terminal cephalopod consensus motif is encoded in two exons, with the last exon containing only the second and third nucleotide of the terminal tryptophan codon followed by a termination codon. This feature is consistently found in all cephalopod lamins analysed so far. For *Nautilus pompilius*, only one type of transcript has been found. In contrast, two transcript variants are present in most Octopodiformes (e.g. *Hapalochlaena maculosa*, *Octopus kaurna*, *O. maya*, *O.vulgaris*) and up to four in the Decapodiformes (e.g. *Chiroteuthis calyx* (3), *Dosidicus gigas* (3), *Euprymna tasmanica* (2), *Octopoteuthis deletron* (3), *Onychoteuthis banksii* (2), *Pterygioteuthis hoylei* (2), *Sepia esculenta* (2), *Sepia pharaonis* (2), *Sepioloidea lineolata* (4), *Vampyroteuthis infernalis* (3), *Watasenia scintillans* (3)). These variants result from alternative splicing of the additional one, respectively two exons, which are located between exon 10 and the two terminal exons (Fig. 3b, c, d, e). Whether with or without the regions encoded by the alternatively spliced exons, all cephalopod lamins terminate with the consensus sQqkkgWlFW (uppercase ≥ 90% identity, lowercase ≥ 50% identity) (Corpet 1988). The C-termini lack negatively charged residues with a few exceptions and are rich in the aromatic residues phenylalanine and tryptophan. Particularly noteworthy is the highly conserved position of two tryptophan residues at the very C-terminus from two points of view. Firstly, this amino acid is one of the rarest in proteins. Secondly, tryptophan has a special role in the interaction of proteins with membranes (Khemaissa et al. 2021).

### CaaX-less lamins in the phylum Annelida

**Fig. 4:**
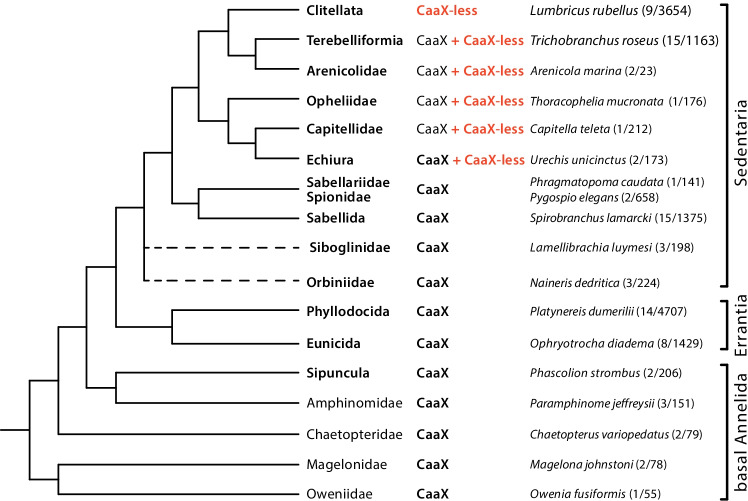
Phylogenetic relationships of 18 clades of the Annelida according to Weigert et al. (2014). Vertical brackets indicate the two major monophyletic clades, Sedentaria and Errantia, as well as the basal annelid taxa. Note that clades of different phylogenetic ranks are shown. The phylogenetic relationship of the Siboglinidae and Orbiniidae is not finally solved (broken lines). CaaX or CaaX-less denotes the presence or absence of the C-terminal CaaX motif in lamin proteins of the respective phyla. CaaX + CaaX-less indicates that both, a lamin with and one without a CaaX motif is expressed. Species name of one representative for each clade is given. The number of species for which lamin sequences were obtained is given in parentheses together with the total number of species of this taxon as listed in the Catalogue of life (Bánki et al. 2022)

Annelida, commonly named as segmented worms, is a morphologically diverse phylum within the Lophotrochozoa. The phylum comprises over 16,000 species, which inhabit a wide range of marine, freshwater and terrestrial habitats and enjoy very different lifestyles. Most basic phylogenetic relationships within the annelids have been clarified in recent years with the help of extensive sequence and phylogenomic analyses (Fig. 4). There are two major clades, the Sedentaria and the Errantia, in addition to several basal groups (Struck et al. 2011, 2015; Weigert et al. 2014; Weigert and Bleidorn 2016). We were able to determine lamin sequences for the 18 clades listed in Fig. 4. Even though the number of lamin sequences obtained from the basal annelid groups is still small, the following picture emerges: Lamins with a C-terminus other than a CaaX motif are found in a subset of clades of the Sedentaria, namely the Clitellata, Terebelliformia, Arenicolidae, Opheliidae, Capitellidae, and Echiura (Fig. 4). The C-termini of all these lamins, similar to those of the cephalopods, lack negative amino acid residues with few exceptions and are rich in aromatic residues (Tables 2, 3, and 4). However, the number of aromatic residues varies between individual lamins (Table 4). This is especially true for the earthworms of the genera Lumbricus, Eisenia, and Glossoscolex, which express two or even three lamins, one of which usually has a particularly high proportion of aromatic residues, as well as for all Terebelliformia that were studied (Table 4).

**Table 3:** Amino acid composition of Cephalopoda lamin C-termini

	Species	C-terminus	aliph	arom	pos	neg	tiny
Cephalopoda	*Octopus kaurna*	TPQKKTWAFW*	0	30	20	0	10
	*Octopus minor*	TPQKKTWAFW*	0	30	20	0	10
	*Octopus vulgaris/sinensis*	TPQKKGWIFW*	10	30	20	0	10
	*Octopus maya*	TPQKKGWIFW*	10	30	20	0	10
	*Enteroctopus megalocyathus*	PSQKKSWIFW*	10	30	20	0	20
	*Hapalochlaena maculosa*	PSQKKSWLFW*	10	30	20	0	20
	*Vampyroteuthis infernalis*	SQQKKGWLFW*	10	30	20	0	20
	*Idiosepius notoides*	SQQKKGWLFW*	10	30	20	0	20
	*Onychoteuthis banksii*	SQQKKGWIFW*	10	30	20	0	20
	*Sthenoteuthis oualaniensis*	SQQKKGWLFW*	10	30	20	0	20
	*Octopoteuthis deletron*	SQQKKGWLFW*	10	30	20	0	20
	*Pterygioteuthis hoylei*	SQQKKSWLFW*	10	30	20	0	20
	*Chiroteuthis calyx*	SQQKKGWLFW*	10	30	20	0	20
	*Watasenia scintillans*	SQQKKGWLFW*	10	30	20	0	20
	*Dosidicus gigas*	SQQKKGWLFW*	10	30	20	0	20
	*Architeuthis dux*	SQQKKGWLFW*	10	30	20	0	20
	*Euprymna tasmanica*	SQQKKGWLFW*	10	30	20	0	20
	*Euprymna scolopes*	SQQKKGWLFW*	10	30	20	0	20
	*Sepiella maindroni*	AQQRKGWLFW*	10	30	20	0	20
	*Sepia pharaonis*	AQQRKGWLFW*	10	30	20	0	20
	*Sepia esculenta*	AQQRKGWLFW*	10	30	20	0	20
	*Sepioloidea lineolata*	QQKKGGWLFW*	10	30	20	0	20
	*Nautilus pompilius*	TGQRRSWLFW*	10	30	20	0	20

Amino acid composition in % of the 10 C-terminal residues of lamins of the indicated species. *aliph*, aliphatic (I, L, V); *arom*, aromatic (F, W, Y); *pos*, positive (K, R, H); neg: negative (D, E); tiny (G, A, S) residues. Since not all 20 amino acids can be assigned to these groups, the sum does not add up to 100% in all cases. *The C-terminus of a polypeptide

**Table 4:** Amino acid composition of alternative lamin C-termini of Annelida and plants

	Species	C-terminus	aliph	arom	pos	neg	tiny
Clitellata	*Helobdella robusta-1*	SSKGWLSFLG*	20	20	10	0	50
	*Helobdella robusta-2*	ASSLITGFFL*	30	20	0	0	40
	*Hirudo medicinalis-1*	GWLSFLSIMG*	30	20	0	0	40
	*Hirudo medicinalis-2*	ASSLITGMFL*	30	10	0	0	40
	*Hirudo verbana-1*	GWLSFLSIMG*	30	20	0	0	40
	*Hirudo verbana-2*	ASSLITGMFL*	30	10	0	0	40
	*Amynthas corticis-1*	TWSLFSIVLK*	40	20	10	0	20
	*Amynthas corticis-2*	RLSSIFNILM*	40	10	10	0	20
	*Amynthas gracilis*	SWSLFSIVLK*	40	20	10	0	30
	*Eisenia fetida-1*	TWSLFSVLLK*	40	20	10	0	20
	*Eisenia fetida-2*	RLSSIFNILM*	40	10	10	0	20
	*Eisenia fetida-3*	WRWSLFSFLK*	20	40	20	0	20
	*Glossoscolex paulistus-1*	WGWPLFGFMK*	10	40	10	0	20
	*Glossoscolex paulistus-2*	SWSLFSVLLK*	40	20	10	0	30
	*Lumbricus castaneus-1*	WRWSLFSFLK*	20	40	20	0	20
	*Lumbricus castaneus-2*	RLSSIFNILM*	40	10	10	0	20
	*Lumbricus rubellus-1*	TWSLFSVLLK*	40	20	10	0	20
	*Lumbricus rubellus-2*	RLSSIFNILM*	40	10	10	0	20
	*Lumbricus rubellus-3*	WRWSLFSFLK*	20	40	20	0	20
Terebelliformia	*Pectinaria gouldii*	NGQRTWFWWN*	0	40	10	0	10
	*Anobothrus sp.*	SRHSWFWWTK*	0	40	30	0	20
	*Amphisamytha carldarei*	GSRSWFWWNK*	0	40	30	0	20
	*Amphicteis gunneri*	GRRSWFWWNK*	0	40	30	0	20
	*Hypania invalida*	KRRSWFFWSK*	0	40	40	0	20
	*Alvinella caudata*	EGRRKWFWWN*	0	40	30	10	10
	*Alvinella pompejana*	EGRRKWFWWN*	0	40	30	10	10
	*Paralvinella fijiensis*	GRRSWFWWNK*	0	40	30	0	20
	*Paralvinella palmiformis*	GRRSWFWWNK*	0	40	30	0	20
	*Paralvinella grasslei*	SRRSWFWWNK*	0	40	30	0	20
	*Melinna oculata*	GRRSWFFWSK*	0	40	30	0	30
	*Eupolymnia crassicornis*	GRRSWFWWNK*	0	40	30	0	20
	*Polycirrus carolinensis*	SESRSWFWWK*	0	40	20	10	30
	*Neoamphitrite robusta*	ARRSWFWWNK*	0	40	30	0	20
	*Thelepus sp.*	SRRSWFWWNK*	0	40	30	0	20
	*Trichobranchus roseus*	SSRRSWFWWN*	0	40	20	0	30
	*Terebellides sp*	SGRGWFFWRN*	0	40	20	0	30
	*Arenicola marina*	NGRSSWLWWR*	10	30	20	0	30
	*Abarenicola pacifica*	ENGRSWLWWR*	10	30	20	10	20
	*Thoracophelia mucronata*	WFPSLFSILK*	30	30	10	0	20
	*Capitella teleta*	WSWSFFSMLR*	10	40	10	0	30
	*Urechis unicinctus*	WGWSFFSVMK*	10	40	10	0	30
	Plant NMCP1 consensus	GKKLWNFLTT*	20	20	20	0	10

Amino acid composition in % of the 10 C-terminal residues of lamins of the indicated species. *aliph*, aliphatic (I, L, V); *arom*, aromatic (F, W, Y); *pos*, positive (K, R, H); *neg*, negative (D, E); tiny (G, A, S) residues. Since not all 20 amino acids can be assigned to these groups, the sum does not add up to 100% in all cases. *The C-terminus of a polypeptide. Plant NMCP 1 consensus from Ciska et al. (2019)

**Fig. 5:**
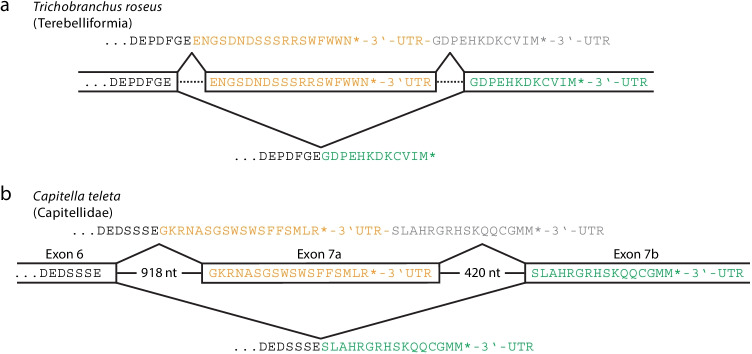
Formation of two lamin mRNA variants by alternative splicing. (a) *Trichobranchus roseus*: formation of the two lamin transcript variants by alternative splicing was inferred from the comparison of the respective transcript variants, which encode the two alternative C-termini together with their corresponding 3′-UTRs (boxes/open boxes). Since no genomic sequences are available for Trichobranchus, intron sizes are not known. Introns are shown as broken lines. Asterisks (*) indicate termination codons. Splicing of both introns results in an mRNA that encodes a lamin with the alternative C-terminus. The resulting protein sequence is shown above the diagram. This mRNA also contains the nucleotide sequence information for the CaaX motif in its 3′-UTR. Of note, this sequence is located downstream of a termination codon and therefore cannot be translated (protein sequence in light grey). Splicing of both introns together with the sandwiched exon results in an mRNA encoding a lamin with a CaaX motif at its C-terminus. The resulting protein sequence is shown below the schematic. (b) *Capitella teleta*: formation of the two lamin splice variants of *Capitella teleta*. The gene structure of the Capitella lamin gene was deduced by comparing the genomic sequences with the sequences of the two transcript variants. The length of the two introns is given as the number of nucleotides (nt)

Of particular interest are observations in the suborder Terebelliformia. We were able to obtain a wealth of lamin transcripts for terebelliformian species due to the availability of extensive transcriptome data originally generated for phylogenetic studies (Stiller et al. 2020). In the majority of the terebellids, we identified up to three transcript variants that differ in the last 10 to 32 triplets of their open reading frames, giving rise to lamin variants with different C-termini. One of the two variants carries a typical CaaX motif, the other a C-terminus, which is rich in aromatic and positively charged residues but lacks aliphatic and negatively charged residues (alternative C-termini). These C-termini meet the sequence consensus gRrSWFwWnK* (uppercase ≥ 90% identity, lowercase ≥ 50% identity) (Corpet 1988). Interestingly, the transcripts encoding the variants with the alternative C-termini contain the sequence information for the corresponding CaaX termini within their 3′-UTR. From this, even though genomic information is missing for Terebellids, the following can be deduced: in the lamin genes of the Terebelliformia, the exon encoding the alternative C-terminus is located upstream of the CaaX-encoding exon and is flanked on both sides by an intron (Fig. 5a). When the two flanking introns are spliced out, the transcript is generated, which encodes the lamin with the alternative C-terminus. The region encoding the CaaX-terminus remains in the 3′-UTR of this mRNA. If, on the other hand, the exon encoding the alternative C-terminus is spliced out together with the two flanking introns, the transcript encoding the CaaX variant is generated (Fig. 5a).

Arenicola is a member of the sister group of the Terebelliformia (Andrade et al. 2015; Helm et al. 2018). Only one transcript was found for *Arenicola marina*. It encodes a lamin with an alternative C-terminus (Table 4), but, like the lamin transcripts of the terebellids, it also contains sequence information for a putative CaaX-terminus in its 3′-UTR. To check whether there exists an mRNA encoding this CaaX variant, the position of the putative splice site was determined by alignment of the Arenicola lamin sequence with those of the terebellids. In this way, the nucleotide sequence of the splice junction could be reconstructed from the transcript and subsequently checked and verified by blast searches using Arenicola RNA-Seq reads.

Capitellidae and Echiura are in a sister group relationship to each other. Together with Opheliidae they form a monophyletic group within Sedentaria (Fig. 4). The lamin sequence of the capitellid *Capitella teleta* has previously been published (Zimek and Weber 2011). It was annotated with an alternative C-terminus. For *Urechis unicictus*, a member of Echiura, in contrast, a lamin with a CaaX terminus was found. Therefore, we paid special attention to the possible existence of two lamin variants within these three groups. Using the approach described above for Arenicola, we were able to detect both lamin variants, one with a C-terminal CaaX motif and one with an alternative C-terminus, for *Capitella teleta* and *Urechis unicincta* as well as for the opheliid, *Thoracophila mucronata*, respectively (Table 4, Supplementary Table S1). For *Ophelina acuminata*, a second member of the Opheliidae, we obtained partial RNA-Seq sequences encoding the corresponding C-terminal regions that closely resemble those of Thoracophila.

For Capitella, the gene structure could be clarified by comparing the genomic sequences with the sequences of the two transcript variants (Fig. 5b). These results support the splicing pattern for the Terebelliformia, which could only be inferred from the comparison of the two transcript variants.

All annelid species in which lamins with alternative C-termini have been found belong to clades that together form a monophyletic group, these groups are the above-discussed Clitellata, Terebelliformia, Arenicolidae, Capitellidae, and Echiura (Weigert et al. 2014; Struck et al. 2015; Weigert and Bleidorn 2016). So far, we have no evidence that any of the species of the Clitellata expresses a CaaX variant in addition to its lamins with alternative C-termini.

In all other annelid groups studied, we found lamins carrying a CaaX motif and so far have no evidence that these species also express lamins variants with alternative C-termini (Fig. 4). For representatives of the Errantia, in particular for the Sabellida, Phyllodocida and Eunicida, we had access to a comparably large amount of transcriptome data as for the Terebelliformia (Laumer et al. 2019; Stiller et al. 2020; Tilic et al. 2022) (Fig. 4). So, if they were present, we should have picked up lamin variants with alternative C-termini in these groups. Far less sequence data are available for the basal annelid groups. The detection of lamins in some of these taxa is currently based on only one or two species.

## Discussion

The search for lamin proteins in the Lophotrochozoa lineage has revealed that lamins with an alternative C-terminus in place of a CaaX motif occur in several branches of this clade. The branches occupy widely different ranks within the phylogenetic tree of the Lophotrochozoa. Lamins with alternative C-termini were found in all four classes of the Rotifera. In molluscs they are restricted to one of the eight classes, the cephalopods, and within annelids they are present in some subgroups of the Sedentaria, but not in others. A reliable identification of lamins with alternative C-termini requires lamin transcripts with a full length ORF, and/or a sufficient number of RNA-Seq reads with which gene annotations can be verified. Since lamin transcripts are only moderately abundant, retrieving a transcript with a full length ORF requires high sequence coverage. Although the amount of sequence information is constantly growing due to intensive phylogenetic studies and due to new sequencing methods, sequence coverage of individual lophotrochozoan groups remains limited at present. Therefore, it cannot be ruled out that lamins with alternative C-termini will be discovered in further lophotrochozoan subgroups as sequencing progresses.

It is assumed that a lamin with its characteristic features including the C-terminal CaaX isoprenylation motif was already present in the last eukaryotic ancestor and was subsequently lost in particular eukaryotic branches (Krüger et al. 2012; Kollmar 2015; Koreny and Field 2016). The most plausible assumption is therefore that lamins with alternative C-termini have evolved independently several times in the lophotrochozoan lineage.

The CaaX motif is a target for post-translational modifications, which, as experimentally proven, is necessary for the association of CaaX proteins with membranes (Clarke 1992). Ablation of the CaaX motif or mutation of the CaaX cysteine residue disables membrane association of lamins (McKeon 1991; Hennekes and Nigg 1994; Hofemeister et al. 2000). The lamins described here do not differ from the CaaX lamins in any other aspect apart from their alternative C-termini. It can also be assumed that these lamins do associate with the inner nuclear membrane and form a lamin filament network like the CaaX lamins. In this context, it is worth noting that a very prominent fibrous nuclear lamina was described as early as 1963 in neurons of the leech, *Hirudo medicinalis* (Gray and Guillery 1963) a species that possesses lamins with alternative C-termini (Table 4). It is therefore reasonable to assume that the alternative C-termini also have functional significance for membrane association.

The alternative lophotrochozoan C-termini can be grouped in two types. C-termini of bdelloid lamins are rich in aliphatic amino acid residues but lack aromatic residues. Most of the others, namely those of the other rotifers, the cephalopods, and the alternative C-termini of the annelids stand out for the absence of negatively charged residues and their high proportion of aromatic residues, in particular phenylalanine and tryptophan.

The nuclear matrix constituent proteins (NMCPs) are the structural components of the plant lamina. They are considered to be the analogs of lamins in plants (Ciska and de la Espina 2013). However, they are not members of the IF protein family but have other evolutionary roots. Type 1 NMCPs (NMCP1) possess highly conserved C-terminal sequences (Ciska et al. 2019). These are strikingly similar to the C-termini of many of the lophotrochozoan lamins discussed here (Tables 3 and 4). Even though the underlying molecular mechanisms are still unknown, the high sequence similarity makes a functional relationship seem more plausible than a purely coincidental sequence match. Tryptophan is the amino acid with the largest side chain with unique physicochemical properties. It is one of the rarest amino acids in proteins. In integral membrane proteins tryptophan is especially located at the level of the water/bilayer interface and plays a role in membrane protein stabilisation and anchoring (Khemaissa et al. 2021).

However, information on the role of tryptophan and phenylalanine residues in proteins other than integral membrane proteins is scarce. Experiments with the Rho guanine exchange factor TGAT may provide a clue, albeit an indirect one. It was shown that the 15-residue long C-terminus of TGAT, which contains one tryptophan, and four phenylalanine residues in addition to two cysteine residues, mediates association with the plasma membrane even in the absence of palmitoylation of the cysteine residues (van Unen et al. 2018). Therefore, it is suggestive that the tryptophan and phenylalanine residues in the lamin C-termini have a function in membrane association. However, this requires experimental proof, which cannot be provided by the authors within the framework of this work. It is hoped that colleagues who have the experimental facilities will take up this question.

### Terebelliformia provide a glance at the playground of evolution

Results of subgroups within the Annelida, namely the Terebelliformia, Arenicolidae, Opheliidae, Capitellidae, and Echiura are of particular interest with respect to the evolution of lamins with alternative C-termini. They point a way to how the switch from lamins carrying a CaaX motif to lamins with alternative C-termini may have occurred. The following scenario can be imagined: In a first step a lamin gene acquired an additional exon encoding a novel C-terminus. Alternative splicing of the corresponding primary transcript produces two lamin mRNAs, which then give rise to two lamin protein variants, one carrying a CaaX motif, the other a new alternative C-terminus. The new C-terminus does not necessarily have to confer membrane association right from the beginning, as the corresponding lamin is co-expressed together with the CaaX lamin and will be targeted to the nuclear membrane by co-transport. Once the alternative C-terminus itself has acquired the property of mediating membrane association, the CaaX motif is dispensable and may be lost. The co-existence of two lamins, products of a single gene, one with an alternative C-terminus, one still carrying a CaaX motif at its C-terminus, is seen so far only in five clades of the Annelida, namely in the Terebelliformia, Arenicolidae, Opheliidae, in Capitella and in Urechis. The fact that mRNAs for both lamin variants could be detected in all cases suggests that both protein variants are indeed co-expressed. In contrast, the Clitellata as well as members of other Lophotrochozoa clades, the Rotifera and the Cephalopoda, have lost the information for a CaaX-carrying lamin. In these organisms, the association of lamins with the nuclear membrane must occur in a different way. A role of the new C-terminal sequences seems obvious, but must be clarified experimentally.

In other cases where lamins exist without a CaaX motif, they are expressed together with a second, constitutively expressed CaaX lamin and, as shown experimentally, are unable to associate with the nuclear membrane on their own (Krohne et al. 1989). In contrast to the cases described for the annelids, these lamins are encoded by separate genes. The first step in the evolution of these CaaX-less lamins was therefore probably a duplication of the lamin gene, followed by differentiation and loss of the CaaX motif.

The appearance of alternative lamin C-termini in clades of different phylogenetic position raises the question of whether they are the result of genetic drift and/or what evolutionary advantage the acquisition of these new C-termini might confer. However, discussion of these issues requires experimental studies that provide information about the nature and strength of the membrane affinity conferred by the alternative C-termini.

We focused our analysis on the lophotrochozoan lineage, as this group was the only one in which lamins with exclusively alternative C-termini were found so far. The number of lophotrochozoan species studied is limited by the sequence information currently available. For many taxa this number is small compared to the number of species for the respective group (Bánki et al. 2022). Moreover, whether lamins with alternative C-termini are actually absent in those groups where they have not been found, or have merely remained undiscovered, must remain open. The C-terminal sequences to be searched for are short and show variability. Negative results in database searches therefore cannot make any definitive statements about their absence. Therefore, it cannot be excluded that further examples of lamins with alternative C-termini will be found within the lochotrophozoan lineage. Likewise, it must remain open whether this is a peculiarity of the Lophotrochozoa. So far, no cases are known in other groups. However, since the occurrence within the Lophotrochozoa is restricted to individual systematic groups of different phylogenetic ranks, very extensive sequence data are needed to make reliable statements for other groups. The Earth BioGenome Project 2020, which aims to sequence all known eukaryotic species in a 10-year timeframe (Lewin et al. 2018), and in particular comprehensive transcriptome data will be able to provide answers here.

## Supplementary Information

Below is the link to the electronic supplementary material.

Supplementary file1 (PDF 178 KB)
