# Supplementary material for: CaaX-less lamins: Lophotrochozoa provide a glance at the playground of evolution
Source: Protoplasma. 2022 Sep 14;260(3):741–56. doi: 10.1007/s00709-022-01809-3 (PMC10125929; doi:10.1007/s00709-022-01809-3)
Supplement: Supplementary file 1 — Supplementary file1 (PDF 178 KB) [file 709_2022_1809_MOESM1_ESM.pdf]

Supplementary Table S1 / CaaX-less lamins: Lophotrochozoa provide a glance at the playground of evolution / Reimer Stick* and Annette Peter / Department of Cell Biology, University of Bremen, P.O. Box 33 04 40, 28334 Bremen, Germany / *Corresponding author at: Department of Cell Biology, University of Bremen, P.O. Box 330440, 28334 Bremen, Germany. / Email address: stick[at]uni-bremen.de / ORCID iD: 0000-0001-5559-5935

	Phylum	Class	Subclass	Infraclass	Order	Family	Species	NCBI:txid	# of lamins	Gene	# aa	ORF com-plete	Accession	CaaX	alt. C-term
1.	Annelida	Clitellata	Hirudinea		Hirudinida	Hirudinidae	***Hirudo medicinalis***	txid6421	2	no	601/589	yes/yes	CAGKPE010009502 / GBRF01045227 / GBRF01013143 / CAGKPE010010645 / GBRF01001119	no/no	INQSRGSGKGWLSFLSIMG*/ITRR GASSLITGMFL*
2.	Annelida	Clitellata	Hirudinea		Hirudinida	Hirudinidae	***Hirudo verbana***	txid311461	2	no	601/586	yes/yes	GGIQ01008227 / GGIQ01064305	no/no	INQSRGSGKGWLSFLSIMG*/ITRR GASSLITGMFL*
3.	Annelida	Clitellata	Hirudinea		Rhynchobdellida	Glossiphoniidae	***Helobdella robusta***	txid6412	2	yes	613/603	yes/yes	JN633937 / AMQM01005840 / AMQM01001041	no/no	VLQSKNSSKGWLSFLG*/SLSRVGA LTRRGASSLITGFFL*
4.	Annelida	Clitellata	Oligochaeta		Crassiclitellata	Glossoscolecidae	***Glossoscolex paulistus***	txid1046353	2	no	622/624	yes/yes	GBIL01086023 / GBIL01068204	no/no	ATKKAGQPKGRRWGWPLFGFMK*/D QQPASSSRGAASRWKSWSLFSVLLK *
5.	Annelida	Clitellata	Oligochaeta		Crassiclitellata	Lumbricidae	***Eisenia fetida***	txid6396	1	yes	619	yes	SACV01008230 / SACV01259777 / CYRZ010210413 / CYRZ010638733 / CYRZ010008257 / SACV01209205 / CYRZ010008257 / SACV01289478 / CYRZ010515727 / CYRZ011558323 / SRA:DRR023799.23135080 / SRA:DRR023799.23642219	no	DEQQAASGSKGGASRWKTWSLFSVL LK*
6.	Annelida	Clitellata	Oligochaeta		Crassiclitellata	Lumbricidae	***Lumbricus castaneus***	txid81491	2	no	615/511	yes/no	GGRB01007498 / GGRB01039505	no/no	SAKKAQQKSPSKWRWSLFSFLK*/S ATDDRLSHNSSWRLSSIFNILM*
7.	Annelida	Clitellata	Oligochaeta		Crassiclitellata	Lumbricidae	***Lumbricus rubellus***	txid35632	1	no	622/27/16	yes	GIFV01067613 / GIFV01096323/GIFV01139202	no	DDQQPASASKGASSRWKTWSLFSVL LK* / DDRLSHNSSWRLSSIFNILM* / AQQKSPSKWRWSLFSFLK*
8.	Annelida	Clitellata	Oligochaeta		Crassiclitellata	Megascolecidae	***Amynthas corticis***	txid351238	1	yes	608	yes	FXXH013155053/FXXH010206241 / FXXG010188656/FXXH010461624 / FXXG010267521/FXXH010432943 / FXXH010454206 / alternative C-term FXXH012891444	no	VASASKGSSSKWKTWSLFSIVLK*/ NLSRNSSWRLSSIFNILM*
9.	Annelida	Clitellata	Oligochaeta		Crassiclitellata	Megascolecidae	***Amynthas gracilis***	txid301145	1	no	620	yes	GIKF01103236.1	no	VASASKGSSSKWKSWSLFSIVLK*
10.	Annelida	Clitellata	Oligochaeta		Enchytraeida	Enchytraeidae	***Enchytraeus crypticus***	txid913645	1(3)	no	533/?/?	no	GALF01009171 / GALF01016831 / GALF01018164	?	?
11.	Annelida	Clitellata	Oligochaeta		Haplotaxida	Naididae	***Olavius algarvensis***	txid188229	1	no	-422	no	HACZ01056262	?	
12.	Annelida	Polychaeta	Echiura		Echiuroidea	Bonelliidae	***Bonellia viridis***	txid47118	1	no	596/	yes	assembly from RNA-Seq SRX5588178	CTVM*	WGWSLFSVLY*
13.	Annelida	Polychaeta	Echiura		Xenopneusta	Urechidae	***Urechis unicinctus***	txid6432	2	no	600/ 602	yes	TRINITY_DN195366 Park et al., 2018	CVVM*	QGEDRGHGSRSGNWGWSFFSVMK*
14.	Annelida	Polychaeta	Errantia		Eunicida	Amphinomidae	***Hermione carunculata***	txid259844	1	no	591	yes	SRA SRX194586	CAIM*	
15.	Annelida	Polychaeta	Errantia		Eunicida	Amphinomidae	***Paramphinome jeffreysii***	txid222009	1	no	588	yes	Launer et al.,2019 ANNE_Pamp_cds.c46014	CVVM	
16.	Annelida	Polychaeta	Errantia		Eunicida	Amphinomidae	***Pareurythoe californica***	txid1633897	1	no	592	yes	assembly from RNA-seq SRX965727	CRVV*	
17.	Annelida	Polychaeta	Errantia		Eunicida	Diurodrilidae	***Diurodrilus subterraneus***	txid1318637	1	no	635	yes	Launer et al.,2019 ANN_Diur_cds.comp155117	CSIM	
18.	Annelida	Polychaeta	Errantia		Eunicida	Dorvilleidae	***Ophryotrocha diadema***	txid169117		no	131	**no**	GENQ01004016 / GENQ01019935	CLIM*	
19.	Annelida	Polychaeta	Errantia		Eunicida	Lumbrineridae	***Ninoe nigripes***	txid318790	1	no	626	yes	Tilic et al.,2022 Mol Phylogenet Evol 166	CSIM*	
20.	Annelida	Polychaeta	Errantia		Eunicida	Oenonidae	***Arabella***	txid65493	1	no	612	yes	Tilic et al.,2022 Mol Phylogenet Evol 166	CFLM*	
21.	Annelida	Polychaeta	Errantia		Eunicida	Onuphidae	***Diopatra cuprea***	txid398472	1	no	616	yes	Tilic et al.,2022 Mol Phylogenet Evol 166	CFLM*	
22.	Annelida	Polychaeta	Errantia		Phyllodocida	Aphroditidae	***Laetmonice cf. iocasica***	txid2781578	1	no	609	yes	Tilic et al.,2022 Mol Phylogenet Evol 166	CSIM*	
23.	Annelida	Polychaeta	Errantia		Phyllodocida	Glyceridae	***Glycera dibranchiata***	txid6350	1	no	601	yes	GASB01024508	CVLM	
24.	Annelida	Polychaeta	Errantia		Phyllodocida	Hesionidae	***Amphiduros pacificus***	txid1209993	1	no	606	yes	Tilic et al.,2022 Mol Phylogenet Evol 166	CSIM*	
25.	Annelida	Polychaeta	Errantia		Phyllodocida	Nephtyidae	***Nephtys hombergii***	txid36121	1	n	604	yes	Tilic et al.,2022 Mol Phylogenet Evol 166	CVVM*	

: Stopp codon; (CaaX): indicates that alternatively spliced transcripts encoding lamins with alternative C-termini contain in addition the sequence information for a CaaX terminus within their 3' UTR.

	Phylum	Class	Subclass	Infraclass	Order	Family	Species	NCBI:txid	# of lamins	Gene	# aa	ORF complete	Accession	CaaX	alt. C-term
26.	Annelida	Polychaeta	Errantia		Phyllodocida	Nereididae	***Alitta virens***	txid880429	1	no	589	yes	GINI01065852	CILQ*	
27.	Annelida	Polychaeta	Errantia		Phyllodocida	Nereididae	***Perinereis aibuhitensis***	txid126650	1	no	590	yes	GDAF01000766	CFLM*	
28.	Annelida	Polychaeta	Errantia		Phyllodocida	Nereididae	***Platynereis dumerilii***	txid6359	1	no	591	yes	GBZT01001351	CILQ*	
29.	Annelida	Polychaeta	Errantia		Phyllodocida	Pholoidae	***Pholoe baltica***	txid318818	1	no	579/93	yes	Tilic et al.,2022 Mol Phylogenet Evol 166	CSIM*	
30.	Annelida	Polychaeta	Errantia		Phyllodocida	Phyllodocidae	***Phyllodoce medipapillata***	txid868040	1	no	599	yes	Tilic et al.,2022 Mol Phylogenet Evol 166	CIVM*	
31.	Annelida	Polychaeta	Errantia		Phyllodocida	Pilargidae	***Pilargis verrucosa***	txid1818081	1	no	610	yes	Tilic et al.,2022 Mol Phylogenet Evol 166	CVIM*	
32.	Annelida	Polychaeta	Errantia		Phyllodocida	Polynoidae	***Harmothoe imbricata***	txid61848	1	no	618	yes	GGLP01024036	CSIM	
33.	Annelida	Polychaeta	Errantia		Phyllodocida	Polynoidae	***Lepidonotopodium***	txid167794	1	no	607/13/14	yes	Tilic et al.,2022 Mol Phylogenet Evol 166	CSIM*	
34.	Annelida	Polychaeta	Errantia		Phyllodocida	Syllidae	***Syllis gracilis***	txid418370	2	no	601/612	yes	Tilic et al.,2022 Mol Phylogenet Evol 166	CSIM*	
35.	Annelida	Polychaeta	Errantia		Phyllodocida	Tomopteridae	***Tomopteris***	txid397573	1	no	598	yes	Tilic et al.,2022 Mol Phylogenet Evol 166	CSIM*	
36.	Annelida	Polychaeta	Sedentaria	Canalipalpata	Sabellida	Fabriciidae	***Fabricinuda trilobata***	txid1002764	1	no	596	yes	Tilic et al., 2020_gene:41717.1/isoform:1	CSIM*	
37.	Annelida	Polychaeta	Sedentaria	Canalipalpata	Sabellida	Fabriciidae	***Manayunkia occidentalis***	txid2704156	1	no	599	yes	Tilic et al., 2020_c27886_g1_i1	CSIM*	
38.	Annelida	Polychaeta	Sedentaria	Canalipalpata	Sabellida	Fabriciidae	***Novafabricia brunnea***	txid1002769	1	no	600	yes	Tilic et al., 2020_c32393_g2_i2	CSLM*	
39.	Annelida	Polychaeta	Sedentaria	Canalipalpata	Sabellida	Sabellariidae	***Phragmatopoma caudata***	txid343980	1	no	611	yes	assembly from RNA-Seq SRX2996542-7	CVMM*	
40.	Annelida	Polychaeta	Sedentaria	Canalipalpata	Sabellida	Sabellidae	***Acromegalomma coloratum***	txid2733411	1	no	596	yes	Tilic et al., 2020_gene:34478.4/isoform:1	CAIM*	
41.	Annelida	Polychaeta	Sedentaria	Canalipalpata	Sabellida	Sabellidae	***Bispira melanostigma***	txid754251	1	no	599/611	yes	Stiller et al., 2020 _c41194_g6_i5/_i7	CLIM*	
42.	Annelida	Polychaeta	Sedentaria	Canalipalpata	Sabellida	Sabellidae	***Bispira turneri***	txid2733419	2	no	600/612	yes	Tilic et al., 2020_gene:46061	CVLM*	
43.	Annelida	Polychaeta	Sedentaria	Canalipalpata	Sabellida	Sabellidae	***Branchiomma conspersum***	txid2733426	1	no	600/612	yes/no	Stiller et al., 2020 _c42611_g1_i2/_i1_li3	CLIM*	
44.	Annelida	Polychaeta	Sedentaria	Canalipalpata	Sabellida	Sabellidae	***Eudistylia vancouveri***	txid6364	1	no	471+117	no	Tilic et al., 2020_gene:27909	CMIM*	
45.	Annelida	Polychaeta	Sedentaria	Canalipalpata	Sabellida	Sabellidae	***Hypsicomus***		1	no	599	yes	Tilic et al., 2020_c50182_g1_i1	CSLM*	
46.	Annelida	Polychaeta	Sedentaria	Canalipalpata	Sabellida	Sabellidae	***Myxicola***	txid76263	1	no	608/598	yes/yes	Stiller et al., 2020 _agalma/gene:53435.2	CIVM*	
47.	Annelida	Polychaeta	Sedentaria	Canalipalpata	Sabellida	Sabellidae	***Pseudopotamilla (oculatum)***	txid279638	1	no	598/608	yes	Stiller et al., 2020 _c41683_g1_i1/_i2	CLIM*	
48.	Annelida	Polychaeta	Sedentaria	Canalipalpata	Sabellida	Sabellidae	***Sabellastarte magnifica***	txid389514	1	no	600	yes	Stiller et al., 2020 _c29287_g1_i1	CLIM*	
49.	Annelida	Polychaeta	Sedentaria	Canalipalpata	Sabellida	Serpulidae	***Romanchella perrieri***	txid2555682	2	no	609/562	yes/no	HBVW01034317/	CSIM/CSIM	
50.	Annelida	Polychaeta	Sedentaria	Canalipalpata	Sabellida	Serpulidae	***Spirobranchus lamarcki***	txid2082999	1	no	611	yes	GGGS01229327	CAIM*	
51.	Annelida	Polychaeta	Sedentaria	Canalipalpata	Sabellida	Serpulidae	***Spriobranchus***	txid344940	1	no	595	yes	Tilic et al., 2020_gene:41159.1/isoform:1	CVIM*	
52.	Annelida	Polychaeta	Sedentaria	Canalipalpata	Sabellida	Siboglinidae	***Lamellibrachia luymesi***	txid238240	1	yes	588	yes	SDWI01000178	CSLM*	
53.	Annelida	Polychaeta	Sedentaria	Canalipalpata	Sabellida	Siboglinidae	***Lamellibrachia satsuma***	txid104711	1	no	588	yes	GEHO01210268	CSLM*	
54.	Annelida	Polychaeta	Sedentaria	Canalipalpata	Sabellida	Siboglinidae	***Paraescarpia echinospica***	txid2080241	1	no	588	yes	GHDL01068126	CSLM*	
55.	Annelida	Polychaeta	Sedentaria	Scolecida	Scolecida	Arenicolidae	***Abarenicola pacifica***	txid273052	1	no	613	yes	assembly from RNA-Seq SRX7658526	CVIM*	ENGRSWLWWR*
56.	Annelida	Polychaeta	Sedentaria	Scolecida	Scolecida	Arenicolidae	***Arenicola marina***	txid6344	1	no	615	yes	GJHO01025586	CVIM*	SGDSDNGRSSWLWWR*
57.	Annelida	Polychaeta	Sedentaria	Scolecida	Scolecida	Capitellidae	***Capitella teleta***	txid283909	1	yes	606	yes	AMQN01009294	no	GKRNASGSWSWSFFSMLR*
58.	Annelida	Polychaeta	Sedentaria		Scolecida	Orbiniidae	***Leitoscoloplos robustus***	txid645995	1	no	603	yes	assembly from RNA-Seq SRX5588175	CVIM*	
59.	Annelida	Polychaeta	Sedentaria		Scolecida	Orbiniidae	***Naineris dendritica***	txid273009	1	no	593	yes	assembly from RNA-Seq SRX1024019	CSIM*	
60.	Annelida	Polychaeta	Sedentaria		Scolecida	Orbiniidae	***Scoloplos amiger***	txid46605	1	no	557	no	assembly from RNA-Seq SRX2848069	CVIM*	
61.	Annelida	Polychaeta	Sedentaria	Canalipalpata	Spionida	Spionidae	***Pygospio elegans***	txid51279	1	no	625	yes	GFPL01004180	CAIM*	
62.	Annelida	Polychaeta	Sedentaria	Canalipalpata	Spionida	Spionidae	***Streblospio benedicti***	txid95538	1	no	468	no	GDBG01126335	CMIM*	
63.	Annelida	Polychaeta	Sedentaria	Canalipalpata	Terebellida	Alvinellidae	***Alvinella caudata***	txid36105	2	no	381(383	no	Stiller et al., 2020 _c14202_g2_i1/g2_i2	CIIM*	EGRDSEGRDSEGRRKWFWWN*
64.	Annelida	Polychaeta	Sedentaria	Canalipalpata	Terebellida	Alvinellidae	***Alvinella pompejana***	txid6376	1	no	607	yes	Stiller et al., 2020 _c19004_g1_i1	(CVIM*)	EGRDSEGRDSEGRRKWFWWN*
65.	Annelida	Polychaeta	Sedentaria	Canalipalpata	Terebellida	Alvinellidae	***Paralvinella fijiensis***	txid36110	1	no	617/627	yes	Stiller et al., 2020 _c33371_g1_i1/_i2/_i3	CTIM*	ERSESEGRDSDSGRRSWFWWNK* splice var.
66.	Annelida	Polychaeta	Sedentaria	Canalipalpata	Terebellida	Ampharetidae	***Amphicteis_cf_gunneri***	txid2019424	1(2)	no	618/626	yes	Stiller et al., 2020 c39174_g1_i1/_g1_i2	CIIM*	EGRDSEGRDSQNGRRSWFWWNK*
67.	Annelida	Polychaeta	Sedentaria	Canalipalpata	Terebellida	Ampharetidae	***Anobothrus sp***	txid194192	1(2)	no	?/?	no	Stiller et al., 2020 _c35143_g1_i1/	CVIQ*	ERDSEDPESGSRHSWFWWTK*
68.	Annelida	Polychaeta	Sedentaria	Canalipalpata	Terebellida	Ampharetidae	***Hypania invalida***	txid1977848	1	no	612	yes	Stiller et al., 2020_c16123_g1_i3	CVIM*	GSQKRRSWFFWSK* splice var.
69.	Annelida	Polychaeta	Sedentaria	Canalipalpata	Terebellida	Melinnidae	***Melinna oculata***	txid2716577	1	no	613	yes	Stiller et al., 2020 _c37598_g1_i1	(CVIM*)	EDRDSEGRRSWFFWSK*

: Stopp codon; (CaaX): indicates that alternatively spliced transcripts encoding lamins with alternative C-termini contain in addition the sequence information for a CaaX terminus within their 3' UTR.

	Phylum	Class	Subclass	Infraclass	Order	Family	Species	NCBI:txid	# of lamins	Gene	# aa	ORF com-plete	Accession	CaaX	alt. C-term
70.	Annelida	Polychaeta	Sedentaria	Canalipalpata	Terebellida	Pectinariidae	***Pectinaria gouldii***	txid260746	1	no	342	no	Stiller et al., 2020 _c30086_g1_i2	CTIM*	NGQRTWFWWN*
71.	Annelida	Polychaeta	Sedentaria	Canalipalpata	Terebellida	Terebellidae	***Amphisamytha carldarei***	txid1311522	1	no	623	yes	Stiller et al., 2020 _c32676_g1_i1/_i2	(CVIM*)	EGRDSEGRDSNSGSRSWFWWNK*
72.	Annelida	Polychaeta	Sedentaria	Canalipalpata	Terebellida	Terebellidae	***Eupolymnia crassicornis***	txid2716571	2	no	603/624	yes	Stiller et al., 2020 c27732_g1_i1/-i2/_i3/_i4	CRIM*	VGSEHIFHQQDRRDSDRESSEGRRSWFWWNK*
73.	Annelida	Polychaeta	Sedentaria	Canalipalpata	Terebellida	Terebellidae	***Lanice conchilega***	txid41793	1	no	601	yes	Stiller et al., 2020 _c23405_g1_i1	CAIM*	
74.	Annelida	Polychaeta	Sedentaria	Canalipalpata	Terebellida	Terebellidae	***Loimia bermudensis***	txid2716590	1	no	604	yes	Stiller et al., 2020 c33371_g1_i2	CVIM*	
75.	Annelida	Polychaeta	Sedentaria	Canalipalpata	Terebellida	Terebellidae	***Neoamphitrite robusta***	txid868091	1	no	613	yes	Stiller et al., 2020 _c37710_g1_i1/_2	CCCM*	DSRDSRESSEARRSWFWWNK*
76.	Annelida	Polychaeta	Sedentaria	Canalipalpata	Terebellida	Terebellidae	***Thelepus sp***	txid36106	1	no	620	yes	Stiller et al., 2020 _c48821_g1_i1/_i2/_i3	(CIIM*)	SGRDSESRRSWFWWNK*
77.	Annelida	Polychaeta	Sedentaria	Canalipalpata	Terebellida	Trichobranchidae	***Terebellides sp***	txid36114	1	no	592	no	Stiller et al., 2020 _c31467_g1_i1	(CVIM*)	ENGDSGRGWFFWRN*
78.	Annelida	Polychaeta	Sedentaria	Canalipalpata	Terebellida	Trichobranchidae	***Trichobranchus roseus***	txid2248427	1	no	608	yes	Stiller et al., 2020 c30090_g2_i2	CVIM*	ENGSDNDSSSRRSWFWWN*
79.	Annelida	Polychaeta	Sedentaria			Chaetopteridae	***Chaetopterus variopedatus***	txid34590	1	no	599	yes	SRA RNA-seq SRX2848071	CIVM*	
80.	Annelida	Polychaeta	Sedentaria			Chaetopteridae	***Phyllochaetopterus sp.***	txid104707	1	no	606	no	Launer et al.,2019 ANNE_Phyl_cds.c49490/cds.c29447	CIVM	
81.	Annelida	Polychaeta	Sedentaria			Opheliidae	***Thoracophelia mucronata***	txid1280055	1	no	607	yes	SRA RNA-seq SRX1024184	(CAIM*)	WFPSLFSILK*
82.	Annelida	Polychaeta			incerta sedis	Dinophilidae	***Dimorphilus gyrociliatus***	txid2664684	1		571	yes	CAD5118442.1	CSIM	
83.	Annelida	Polychaeta			Sabellida	Oweniidae	***Owenia fusiformis***	txid6347	1	no	604	yes	CAC9669417	CVIM	
84.	Annelida	Polychaeta			Spionida	Magelonidae	***Magelona johnstoni***	txid1436028	1	no	601	yes	Launer et al.,2019 ANNE_Mjoh_c18304	CVIM	
85.	Annelida	Polychaeta			Spionida	Magelonidae	***Magelona pitelkai***	txid1653079	1	no	595	yes	SRA RNA-seq SRX1022769	CVIM*	
86.	Annelida	Polychaeta				Histriobdellidae	***Histriobdella***	txid272734	1	no	628	yes	Tilic et al.,2022 Mol Phylogenet Evol 166	CAIM*	
87.	Annelida	Sipuncula		Sipunculidea	Golfingiida	Phascolionidae	***Phascolion strombus***	txid74861	1	no	590	yes	Kollmar, 2015	CILQ*	
88.	Annelida	Sipuncula		Sipunculidea	Golfingiida	Sipunculidae	***Phascolopsis gouldii***	txid6442	1	no	440	no	Launer et al.,2019 ANNE_Plcs_comp156598/156598	CSIM*	
89.	Brachiopoda	Lingulata			Lingulida	Lingulidae	***Glottidia pyramidata***	txid34515	1	no	596	yes	Launer et al.,2019 BRAC_Gpyr_comp50969	CIIM	
90.	Brachiopoda	Lingulata			Lingulida	Lingulidae	***Lingula anatina***	txid7574	1	yes	591	yes	LFEI02000007_pos1117000-1128000 / GDJY01027244 /comp146393	CIVM*	
91.	Brachiopoda	Rhynchonellata			Rhynchonellida	Hemithyrididae	***Hemithiris psittacea***	txid763142	1	no	597	yes	Launer et al.,2019 BRAC_Hpst_comp29307	CAIM	
92.	Brachiopoda	Rhynchonellata			Terebratulida	Laqueidae	***Terebratalia transversa***	txid34513	1	no	587	yes	Launer et al.,2019 BRAC_Ttvs_comp46003	CVVM	
93.	Bryozoa	Gymnolaemata			Cheilostomatida	Bugulidae	***Bugula neritina***	txid10212	2	yes	589/444	yes/no	VXIV01003054/	CVVM*/CLIM	
94.	Bryozoa	Gymnolaemata			Cheilostomatida	Membraniporidae	***Membranipora membranacea***	txid95170	2	no	586/381	yes/no	Launer et al.,2019 BRYO_Mmem_tri.5765/6148	CSIM/	
95.	Bryozoa	Gymnolaemata			Ctenostomatida	Flustrellidridae	***Flustrellidra corniculata***	txid2565296	2	no	583 /464	yes/no	Launer et al.,2019 BRYO_Fcor_cds.c49799	CSIM/CSIM	
96.	Bryozoa	Phylactolaemata				Cristatellidae	***Cristatella mucedo***	txid67896	1	yes	599	yes	WPIO01000056	CVVM*	
97.	Chaetognatha	Sagittoidea			Aphragmophora	Krohnittidae	***Krohnitta***	txid366401	1	no	496	yes	Marlétaz et al., 2019 Curr Biol 29,312-18	CAIM*	
98.	Chaetognatha	Sagittoidea			Aphragmophora	Sagittidae	***Sagitta elegans***	txid10231	1	no	510	yes	Launer et al.,2019 CHAE_Selg_cds.c45366	CAIM	
99.	Chaetognatha	Sagittoidea			Aphragmophora	Sagittidae	***Sagitta gazellae***	txid52898	1	no	511	yes	Marlétaz et al., 2019 Curr Biol 29,312-18	CVVM*	
100.	Chaetognatha	Sagittoidea			Aphragmophora	Sagittidae	***Sagitta serratodentata***	txid52891	1	no	503	yes	Marlétaz et al., 2019 DN41697_c2_g1-g2	CAIM*	
101.	Chaetognatha	Sagittoidea			Aphragmophora	Sagittidae	***Sagitta setosa***	txid52890	1	no	505	yes	Marlétaz et al., 2019 Curr Biol 29,312-18	CAIM*	
102.	Chaetognatha	Sagittoidea			Phragmophora	Eukrohniidae	***Eukrohnia***	txid52636	1	no	480	no	Marlétaz et al., 2019 Curr Biol 29,312-18	CAIM*	
103.	Chaetognatha	Sagittoidea			Phragmophora	Spadellidae	***Paraspadella gotoi***	txid34758	1	no	488	yes	Marlétaz et al., 2019 Curr Biol 29,312-18	CAIM*	
104.	Chaetognatha	Sagittoidea			Phragmophora	Spadellidae	***Spadella cephaloptera***	txid52888	1	n	493	yes	Marlétaz et al., 2019 Curr Biol 29,312-18	CAIM*	
105.	Cycliophora						***Symbion americanus***	txid358452	1	no	583	yes	assembly from RNA-Seq SRX12009316/SRX1122263	CCVM*	
106.	Cycliophora						***Symbion pandora***	txid69817	1	no	583	yes	Launer et al.,2019 CYCL_SyRN_cds.c18282	CCVM	
107.	Dicyemida					Dicyemidae	***Dicyema_sp 1= D. japonicum***	:txid399803	1	no	539	yes	Zverkov et al., 2019 rontiers in Genetics	CVVM*	
108.	Dicyemida					Dicyemidae	***Dicyema_sp.1***	txid10217?	1		539	yes	Zverkov et al., 2019	CVVM*	
109.	Dicyemida					Dicyemidae	***Dicyema_sp.2***	txid10217?	1		562	yes	Zverkov et al., 2019	CVVM*	
110.	Dicyemida					Dicyemidae	***Dicyema_sp2***		1	no	562	yes	Zverkov et al., 2019 frontiers in Genetics	CVVM*	
111.	Dicyemida					Dicyemidae	***Dicyema_sp3***		1	yes	555	yes	Zverkov et al., 2019 frontiers in Genetics	CVVM*	

: Stopp codon; (CaaX): indicates that alternatively spliced transcripts encoding lamins with alternative C-termini contain in addition the sequence information for a CaaX terminus within their 3' UTR.

	Phylum	Class	Subclass	Infraclass	Order	Family	Species	NCBI:txid	# of lamins	Gene	# aa	ORF com-plete	Accession	CaaX	alt. C-term
112.	Entoprocta					Barentsiidae	***Barentsia gracilis***	txid232741	1	no	142 + 276	no	Launer et al.,2019 ENTO_Bgra_comp55759/comp60659	CRLM	
113.	Entoprocta					Loxosomatidae	***Loxomitra sp.***	txid768837	1	no	117	no	Launer et al.,2019 ENTO_Lxmt_cds.c1962_g1_i1	CSIM*	
114.	Entoprocta					Loxosomatidae	***Loxosoma pectinaricola***	txid768841	1	no	123	no	Launer et al.,2019 ENTO_Loxp_comp62901		
115.	Entoprocta					Pedicellinidae	***Pedicellina sp.***	txid43122	1	no	584	yes	Launer et al.,2019 ENTO_PcIF_cds.c1460_g2_i1	CRLM	
116.	Gastrotricha				Chaetonotida	Chaetonotidae	***Lepidodermella squamata***	txid1194616	1	no	609	yes	Launer et al.,2019 GAST_Lepi_comp395683	CCVM	
117.	Gastrotricha				Chaetonotida	Muselliferidae	***Diuronotus aspetos***	txid1681188	1	no	520	yes	Launer et al.,2019 GAST_Dnot_cds.c59638	CAIM	
118.	Gastrotricha				Macrodasyida	Dactylopodolidae	***Dactylopodola baltica***	txid1035121	1	no	398	no	Struck et al., 2014 (15_6750) Laumer cds.c16781	CIIC*	
119.	Mollusca	Bivalvia	Autobranchia		Arcoida	Arcidae	***Scapharca broughtonii /Anadara b.***	txid148819	1	no	608	yes	GEXI01103917	CSIM*	
120.	Mollusca	Bivalvia	Autobranchia	Euheterodonta	Myida	Dreissenidae	***Dreissena polymorpha***	txid45954	1	no	579	yes	GHIW01002434	CSIM*	
121.	Mollusca	Bivalvia	Autobranchia	Euheterodonta	Myida	Dreissenidae	***Dreissena rostriformis***	txid205083	1	no	579	yes	GHIX01015391	CSIM*	
122.	Mollusca	Bivalvia	Autobranchia		Mytiloida	Mytilidae	***Mytilus edulis***	txid6550	1	no	593	yes	GHII01258557	CAVM*	
123.	Mollusca	Bivalvia	Autobranchia		Mytiloida	Mytilidae	***Mytilus galloprovincialis***	txid29158	1	yes	593	yes	APJB011892108 / LNJA010071440/ LNJA010063673 / LNJA010323405 / LNJA010186080 / GHIK01149465	CAVM*	
124.	Mollusca	Bivalvia	Autobranchia		Mytiloida	Mytilidae	***Perna viridis***	txid73031	1	no	561	**no**	GEKL01052201	?	
125.	Mollusca	Bivalvia	Autobranchia		Ostreida	Ostreidae	***Crassostrea gigas***	txid29159	1	yes	590	yes	AFTI01024655	CSIM*	
126.	Mollusca	Bivalvia	Autobranchia		Ostreida	Ostreidae	***Crassostrea virginica***	txid6565	1	no	579	yes	GGQE01120994	CSIM*	
127.	Mollusca	Bivalvia	Autobranchia		Pectinida	Pectinidae	***Mizuhopecten yessoensis***	txid6573	1	no	601	yes	XM_021501711	CSIM*	
128.	Mollusca	Bivalvia	Autobranchia		Pectinida	Pectinidae	***Nodipecten subnodosus***	txid330909	1	no	602	yes	GFVX01047193	CSVM*	
129.	Mollusca	Bivalvia	Autobranchia		Pectinida	Pectinidae	***Pecten maximus***	txid6579	1	no	602	yes	GGAF01045868	CSIM*	
130.	Mollusca	Bivalvia	Autobranchia		Unionida	Anodontinae	***Cristaria plicata***	txid165446	1	no	588	yes	MolluscDB	CSLM*	
131.	Mollusca	Bivalvia	Autobranchia		Unionida	Unionidae	***Elliptio complanata***	txid55832	1	no	587	yes	GAHW01005570	CSVM*	
132.	Mollusca	Bivalvia	Autobranchia		Unionida	Unionidae	***Villosa lienosa /Leaunio lienosus***	txid2569902	1	no	587	yes	JR507061	CSVM*	
133.	Mollusca	Bivalvia	Autobranchia	Euheterodonta	Venerida	Cyrenidae	***Corbicula fluminea***	txid45949	1	no	579	yes	GFYR01024243	CSIM*	
134.	Mollusca	Bivalvia	Autobranchia	Euheterodonta	Venerida	Veneridae	***Ruditapes decussatus***	txid104385	1	no	581	yes	GFXP01009069	CSIM*	
135.	Mollusca	Bivalvia	Autobranchia	Euheterodonta	Venerida	Veneridae	***Ruditapes philippinarum***	txid129788	1	yes	581	yes	QUSP01000679 / GHAV01578047	CSIM*	
136.	Mollusca	Bivalvia	Autobranchia	Euheterodonta	Venerida	Vesicomyidae	***Calyptogena marissinica***	txid2291877	1	no	583	yes	GIAS01020000	CSIM*	
137.	Mollusca	Bivalvia	Autobranchia	Euheterodonta	Venerida	Vesicomyidae	***Phreagena okutanii***	txid1298646	1	no	583	yes	GIAT01012266	CSIM*	
138.	Mollusca	Caudofoveata			Chaetodermatida	Chaetodermatidae	***Chaetoderma nitidulum***	txid256131	1	no	589	no	SRX2638180 manual assembly	CSIM*	
139.	Mollusca	Caudofoveata			Chaetodermatida	Prochaetodermati[illegible]	***Prochaetoderma californicum***	txid1541961	1	no	608	yes	Struck et al., 2014 comp156308	CSIM*	
140.	Mollusca	Caudofoveata			Limifossorida	Scutopodidae	***Scutopus ventrolineatus***	txid52922	1	no	597	yes	MolluscDB + RSA RNA-Seq	CSIM*	
141.	Mollusca	Cephalopoda	Coleoidea		incertae sedis	Idiosepiidae	***Idiosepius notoides/Xipholeptos***	txid66914	1	no	603	yes	GFNE01043343	no	TPSQQKKGWLFW*
142.	Mollusca	Cephalopoda	Coleoidea		Octopoda	Octopodidae	***Enteroctopus megalocyathus***	txid653772	3	no	596/97/97	yes	GJGG01247034/36/38/	no	TPSQKKSWIFW*
143.	Mollusca	Cephalopoda	Coleoidea		Octopoda	Octopodidae	***Hapalochlaena maculosa***	txid61716	2	yes	593/602	yes	GEXH01102036-7/ octopodresearch.org ScBNHFi_26842	no/no	TPSQKKSWLFW*
144.	Mollusca	Cephalopoda	Coleoidea		Octopoda	Octopodidae	***Octopus bimaculoides***	txid37653	1	(yes)	626	no	LGKD01216286		gene incomplete?
145.	Mollusca	Cephalopoda	Coleoidea		Octopoda	Octopodidae	***Octopus kaurna***	txid243731	2	no	590/599	yes	GEXG01007594-5	no	TPQKKTWAFW*
146.	Mollusca	Cephalopoda	Coleoidea		Octopoda	Octopodidae	***Octopus maya***	txid623738	2	no	594	yes/no	GFAI01044771 / GGXQ01054822	no	TPQKKGWIFW*
147.	Mollusca	Cephalopoda	Coleoidea		Octopoda	Octopodidae	***Octopus minor/Callistoctopus***	txid515824	1	yes	600	yes	octopodresearch.org oct008458F	no	TPQKKTWAF(W)*
148.	Mollusca	Cephalopoda	Coleoidea		Octopoda	Octopodidae	***Octopus vulgaris/sinensis***	txid6645	2	yes	590/599	no	VCDQ01000023 / GGNR01003860-1 / GGNR01010035-6	no	TPQKKGWIFW*
149.	Mollusca	Cephalopoda	Coleoidea		Sepiida	Sepiadariidae	***Sepioloidea lineolata***	txid61742	4	no	[illegible]/600/604/[illegible]	yes	GEXF01054492-95	no	TPQQKKGGWLFW*
150.	Mollusca	Cephalopoda	Coleoidea		Sepiida	Sepiidae	***Sepia esculenta***	txid31210	2	no	608	yes	GGQU01016097-8	no	TPAQQRKGWLFW
151.	Mollusca	Cephalopoda	Coleoidea		Sepiida	Sepiidae	***Sepia pharaonis***	txid158019	2	no	595/607		GEIE01005029-30	no	TPAQQRKGWLFW*

: Stopp codon; (CaaX): indicates that alternatively spliced transcripts encoding lamins with alternative C-termini contain in addition the sequence information for a CaaX terminus within their 3' UTR.

	Phylum	Class	Subclass	Infraclass	Order	Family	Species	NCBI:txid	# of lamins	Gene	# aa	ORF complete	Accession	CaaX	alt. C-term
152.	Mollusca	Cephalopoda	Coleoidea		Sepiida	Sepiidae	***Sepiella maindroni***	txid153280	1	no	607	yes	GFLT01030827	no	TPAQQRKGWLFW*
153.	Mollusca	Cephalopoda	Coleoidea		Sepiolida	Sepiolidae	***Euprymna scolopes***	txid6613	1	(yes)	603	yes	SRIE01022304 / GEOX01052043 / GEOX01007122 / GDAC01000717-18	no	TPSQQKKGWLFW*
154.	Mollusca	Cephalopoda	Coleoidea		Sepiolida	Sepiolidae	***Euprymna tasmanica***	txid70205	2	no	603	yes	GEXE01028173-74	no	TPSQQKKGWLFW*
155.	Mollusca	Cephalopoda	Coleoidea		Teuthida	Architeuthidae	***Architeuthis dux***	txid256136	1	yes	606	yes	VCCN01003977.1	no	TPSQQKKGWLF*
156.	Mollusca	Cephalopoda	Coleoidea		Teuthida	Chiroteuthidae	***Chiroteuthis calyx***	txid559536	3	no	589-608	yes	GGNC01024998/-99/GGNC01025000	no/no/no	TPSQQKKGWLFW*
157.	Mollusca	Cephalopoda	Coleoidea		Teuthida	Enoploteuthidae	***Pterygioteuthis hoylei***	txid559549	3	no	596	yes	GGND01050920-22	no/no/no	TPSQQKKSWLFW*
158.	Mollusca	Cephalopoda	Coleoidea		Teuthida	Enoploteuthidae	***Watasenia scintillans***	txid6625	1	no	95/604/60	yes	GEDX01028016 / GEDX01028018 / GEDW01094765	no	TPSQQKKGWLFW*
159.	Mollusca	Cephalopoda	Coleoidea		Teuthida	Octopoteuthidae	***Octopoteuthis deletron***	txid1582096	3	no	95/607/62	yes	GGNB01050902-4	no/no/no	TPSQQKKGWLFW*
160.	Mollusca	Cephalopoda	Coleoidea		Teuthida	Ommastrephidae	***Dosidicus gigas***	txid346249	3	no	596-608	yes	GHKL01032059-61/GGNG01034845-46	no/no/no	TPSQQKKGWLFW*
161.	Mollusca	Cephalopoda	Coleoidea		Teuthida	Ommastrephidae	***Sthenoteuthis oualaniensis***	txid34553	1	no	605	yes	GHKH01017321	no	TPSQQKKGWLFW*
162.	Mollusca	Cephalopoda	Coleoidea		Teuthida	Onychoteuthidae	***Onychoteuthis banksii***	txid392296	2	no	594/613	yes	GHKK01065758 / GHKK01065757	no/no	TPSQQKKGWIFW*
163.	Mollusca	Cephalopoda	Coleoidea		Vampyromorpha	Vampyroteuthidae	***Vampyroteuthis infernalis***	txid55288	3	no	606/616/?	yes/no	GGNA01077094-96	no	TPSQQKKGWLFW*
164.	Mollusca	Cephalopoda	Nautiloidea		Nautilida	Nautilidae	***Nautilus pompilius***	txid34573	1	no	606	yes	Transcriptome comp1708, Ogura (2013) / SRR027039.115084 / SRR2857280.156904157.2	no	SNTGQRRSWLFW*
165.	Mollusca	Gastropoda	Caenogastropoda		Architaenioglossa	Ampullariidae	***Pomacea canaliculata***	txid400727	1	yes	593	yes	SRJH01000001 / GBZZ01067549	CIIM*	
166.	Mollusca	Gastropoda	Caenogastropoda		Architaenioglossa	Viviparidae	***Cipangopaludina cathayensis***	txid570432	1	no	598	yes	GCEL01089602	CVVM*	
167.	Mollusca	Gastropoda	Caenogastropoda		Architaenioglossa	Viviparidae	***Sinotaia purificata***	txid1134467	1	no	599	yes	GIUB01138152	CVVM*	
168.	Mollusca	Gastropoda	Caenogastropoda		Littorinimorpha	Bithyniidae	***Bithynia siamensis goniomphalos***	txid479249	1		601	yes	GAGS01012184 / SRR768418.23694739.2 / SRR2886788.193613587.1	CAIM*	
169.	Mollusca	Gastropoda	Caenogastropoda		Littorinimorpha	Calyptraeidae	***Crepidula atrasolea***	txid136598	1		596	yes	GFWJ01173774	CVVM*	
170.	Mollusca	Gastropoda	Caenogastropoda		Littorinimorpha	Littorinidae	***Littorina littorea***	txid31216	1	no	602	yes	GGCG01032389	CVVM*	
171.	Mollusca	Gastropoda	Caenogastropoda		Littorinimorpha	Littorinidae	***Littorina saxatilis***	txid31220	1		602	yes	GHUL01033528	CVVM*	
172.	Mollusca	Gastropoda	Caenogastropoda		Littorinimorpha	Naticidae	***Neverita didyma***	txid1027435	1		601	yes	GHHQ01005935	CVVM*	
173.	Mollusca	Gastropoda	Caenogastropoda		Littorinimorpha	Ranellidae	***Charonia lampas***	txid500097	1	no	597	yes	GIQZ01018305	CVVM*	
174.	Mollusca	Gastropoda	Caenogastropoda		Littorinimorpha	Tateidae	***Potamopyrgus antipodarum***	txid145637	1		600	yes	GGFE01005073	CIVM*	
175.	Mollusca	Gastropoda	Caenogastropoda		Neogastropoda	Muricidae	***Rapana venosa***	txid55521	2		600/601	yes	GGVW01056429 / GDIA01147516	CVVM*/CVVM*	
176.	Mollusca	Gastropoda	Caenogastropoda			Semisulcospiridae	***Semisulcospira coreana***	txid364284	1	no	598	yes	GGNX01059597	CVVM*	
177.	Mollusca	Gastropoda	Heterobranchia		Aplysiida	Aplysiidae	***Aplysia californica***	txid6500	1	yes	595	yes	AASC03031649 / GBBG01094938	CFVM	
178.	Mollusca	Gastropoda	Heterobranchia		Pteropoda	Clionidae	***Clione limacina***	txid71516	1	no	590	yes	GESV01011451	CFVM*	
179.	Mollusca	Gastropoda	Heterobranchia		Pteropoda	Limacinidae	***Limacina helicina***	txid220649	1	no	610	yes	GFNM01021768	CSIM*	
180.	Mollusca	Gastropoda	Heterobranchia		Stylommatophora	Arionidae	***Arion vulgaris***	txid1028688	1	no	582	yes	HACG01031047	CRVM*	
181.	Mollusca	Gastropoda	Heterobranchia		Stylommatophora	Bradybaenidae	***Bradybaena similaris***	txid145626	1	no	579	yes	GHAD01178922	CRVM*	
182.	Mollusca	Gastropoda	Heterobranchia		Stylommatophora	Geomitridae	***Candidula unifasciata***	txid1229668	1	no	580	yes	HBUC01053756	CRVM*	
183.	Mollusca	Gastropoda	Heterobranchia		Stylommatophora	Helicidae	***Cepaea nemoralis***	txid28835	1	no	582	yes	GFLU01070413	CRVM*	
184.	Mollusca	Gastropoda	Heterobranchia			Physidae	***Physella acuta***	txid109671	1	no	579	yes	GHAL01015400	CHVM*	
185.	Mollusca	Gastropoda	Heterobranchia			Planorbidae	***Biomphalaria glabrata***	txid6526	1	yes	589	yes	APKA01062750 / APKA01062747 / APKA01111564 / APKA01080626	CMVM*	
186.	Mollusca	Gastropoda	Heterobranchia			Planorbidae	***Biomphalaria pfeifferi***	txid112525	1	no	597	yes	GFMW01153216	CMLM*	
187.	Mollusca	Gastropoda	Neomphalina			Peltospiridae	***Gigantopelta aegis***	txid1735272	1	no	596	yes	XM_041515444	CIIM*	
188.	Mollusca	Gastropoda	Patellogastropoda			Lottiidae	***Lottia gigantea***	txid225164	1	yes	591	yes	AMQO01003898 / FC576543 / FC771163 / FC758996	CAIM*	
189.	Mollusca	Gastropoda	Patellogastropoda			Patellidae	***Patella vulgata***	txid6465	1	no	594	yes	GILA01043554	CAIM*	
190.	Mollusca	Gastropoda	Vetigastropoda		Lepetellida	Haliotidae	***Haliotis discus hannai***	txid42344	1		600	yes	GIGJ01058997	CAIM*	
191.	Mollusca	Gastropoda	Vetigastropoda		Lepetellida	Haliotidae	***Haliotis fulgens***	txid6456	1		600	yes	GGVS01059110	CAIM*	
192.	Mollusca	Gastropoda	Vetigastropoda		Lepetellida	Haliotidae	***Haliotis laevigata***	txid36097	1			no	GFTT01091532	CAIM*	

: Stopp codon; (CaaX): indicates that alternatively spliced transcripts encoding lamins with alternative C-termini contain in addition the sequence information for a CaaX terminus within their 3' UTR.

	Phylum	Class	Subclass	Infraclass	Order	Family	Species	NCBI:txid	# of lamins	Gene	# aa	ORF com-plete	Accession	CaaX	alt. C-term
193.	Mollusca	Gastropoda	Vetigastropoda		Lepetellida	Haliotidae	***Haliotis rubra***	txid36100	1	yes	600	yes	QXJH01000107	CAIM*	
194.	Mollusca	Gastropoda	Vetigastropoda		Lepetellida	Haliotidae	***Haliotis tuberculata***	txid36103	1		597	yes	GEAU01297166	CAIM*	
195.	Mollusca	Monoplacophora			Tryblidiida	Neopilinidae	***Laevipilina hyalina***	txid651133	1	n	595	yes	MolluscDB + SRA RNA-Seq	CVIM*	
196.	Mollusca	Polyplacophora	Neoloricata		Chitonida	Acanthochitonidae	***Acanthochitona crinita***	txid126420	1	no	595	yes	MolluscDB	CSVM*	
197.	Mollusca	Polyplacophora	Neoloricata		Chitonida	Acanthochitonidae	***Acanthochitona fascicularis***	txid55704	1	no	595	yes	GJAX01000309	CSVM*	
198.	Mollusca	Polyplacophora	Neoloricata		Chitonida	Chitonidae	***Acanthopleura granulata***	txid61363	1	yes	592	yes	JABBOT010000005 / Varney etal.,2020 Genome Biol Evol	CAIM*	
199.	Mollusca	Polyplacophora	Neoloricata		Chitonida	Chitonidae	***Chiton olivaceus***	txid256108	1	no	154+392	no	Launer et al.,2019 MOLL_Cton_cds.Locus_19756/31084/43900	CSIM	
200.	Mollusca	Polyplacophora			Lepidopleurida	Lepidopleuridae	***Leptochiton asellus***	txid211853	1	no	598	yes	Struck et al., 2014 comp121304	CSIM*	
201.	Mollusca	Polyplacophora			Lepidopleurida	Lepidopleuridae	***Leptochiton rugatus***	txid413421	1	no	584	yes	Launer & Struck	CFIM*	
202.	Mollusca	Scaphopoda			Dentaliida	Dentaliidae	***Antalis entalis***	txid211836	1	no	597	yes	SRA SRX091287 / SRX2422915	CAIM*	
203.	Mollusca	Scaphopoda			Dentaliida	Dentaliidae	***Graptacme eborea***	txid55752	1	no	589	yes	Struck et al., 2014 comp65739	CAIM*	
204.	Mollusca	Scaphopoda			Dentaliida	Gadilinidae	***Gadila tolmiei***	txid1077242	1		586	yes	MolluscDB Gadila_tolmiei_mdb_srp007793_v1_0__protein__MDBGTOLMPDN74905_c10_g1_i5	CAIM*	
205.	Mollusca	Solenogastres			Cavibelonia	Epimeniidae	***Epimenia babai***	txid231999	1	no	601	yes	WURV01007323.1./WURV01001640.1	CAIM*	
206.	Mollusca	Solenogastres			Cavibelonia	Proneomeniidae	***Proneomenia custodiens***	txid1500391	1	no	595	yes	Launer et al.,2019 MOLL_Pcus_comp67387/62337	CSIM	
207.	Mollusca	Solenogastres			Neomeniamorpha	Neomeniidae	***Neomenia megatrapezata***	txid1077245	1	yes	593	yes	Struck et al., 2014 (9_17836 / 9_53_RC)	CSVM*	
208.	Mollusca	Solenogastres			Pholidoskepia	Gymnomeniidae	***Gymnomenia pellucida***	txid1918950	1	no	593	yes	MolluscDB	CTVM*	
209.	Nemertea	Palaeonemertea				Cephalothricidae	***Cephalotrix linearis***	txid937754	1	no	572	yes	Struck et al., 2014 (14_11692)	CIMM*	
210.	Nemertea	Palaeonemertea				Tubulanidae	***Tubulanus polymorphus***	txid672921	1	no	573	yes	Struck et al., 2014 (22_1421)	CSVM*	
211.	Nemertea	Pilidiophora			Heteronemertea	Lineidae	***Notospermus geniculatus***	txid416868	1	yes	589	yes	NMRB01000700 / GFRY01032064	CAIM*	
212.	Phoronida						***Phoronis australis***	txid115415	1		582	yes	GFSC01007597	CVVM*	
213.	Phoronida						***Phoronis psammophila***	txid67897	1	no	585	yes	Launer et al.,2019 PHOR_Ppsa_comp73191	CVIM	
214.	Phoronida						***Phoronopsis harmeri***	txid490051	1	no	582	yes	Launer et al.,2019 PHOR_Phar_cds.c145698	CSIM	
215.	Platyhelminthes	Cestoda	Eucestoda		Cyclophyllidea	Hymenolepididae	***Hymenolepis diminuta***	NCBI:txid621	2	yes	551/585	yes/yes	UYSG01000199	CSLM*/CALM*	
216.	Platyhelminthes	Cestoda	Eucestoda		Cyclophyllidea	Hymenolepididae	***Hymenolepis microstoma***	txid85433	2	yes	581/587	yes/yes	Kollmar, 2015 / CDS25469 / CDS25470/71	CSLM*/CALM*	
217.	Platyhelminthes	Cestoda	Eucestoda		Cyclophyllidea	Taeniidae	***Echinococcus granulosus***	txid6210	2	yes	583/591	yes/yes	XP_024353629 / XP_024353630	CSLM*/CVLM*	
218.	Platyhelminthes	Cestoda	Eucestoda		Cyclophyllidea	Taeniidae	***Echinococcus multilocularis***	txid6211	2	yes	583/596	yes/?	CBLO020001216	CSLM*/CVLM*	
219.	Platyhelminthes	Cestoda	Eucestoda		Cyclophyllidea	Taeniidae	***Taenia asiatica***	txid60517	1		584	yes	LWMJ02000031	CSLM*	
220.	Platyhelminthes	Cestoda	Eucestoda		Cyclophyllidea	Taeniidae	***Taenia multiceps***	txid94034	2	yes	584/591		MRSW03000001 / JR934635 / JR929963	CSLM*/CVLM*	
221.	Platyhelminthes	Rhabditophora			Macrostomida	Macrostomidae	***Macrostomum lignano***	txid282301	1/?	yes	663/668	yes/yes	NIVC01002033 / GEXL01003443	CSIM*/CSVM*	
222.	Platyhelminthes	Rhabditophora			Macrostomida	Macrostomidae	***Macrostomum tuba***	txid52054	2	yes	690/?	yes/no	GFJZ01065020 / GFJZ01078832 / GFJZ01073517 / GFJZ01034354	CRLM*/CCVM*	
223.	Platyhelminthes	Rhabditophora			Tricladida	Dendrocoelidae	***Bdellocephala annandalei***	txid1421413	2	no	543/?	yes/no	FX808606/FX829847 TSA	CSIM*	
224.	Platyhelminthes	Rhabditophora			Tricladida	Dendrocoelidae	***Proctotyla fluviatilis***	txid231627	3		612/526/?	yes/yes/no	aus Kollmar, 2015	CSLM/CSMM/?	
225.	Platyhelminthes	Rhabditophora			Tricladida	Dugesiidae	***Dugesia japonica***	txid6161	1	yes	615	yes	IAAB01059110/GFJY01021562	CSLM*	
226.	Platyhelminthes	Rhabditophora			Tricladida	Dugesiidae	***Schmidtea mediterranea***	txid79327	1	yes	617/495	yes	AUVC01026413 / GFPD01043123 // AAWT01019235 / GFPD01036671	CSMM*	
227.	Platyhelminthes	Trematoda	Digenea		Plagiorchiida	Fasciolidae	***Fasciola hepatica***	txid6192	1	no	593	yes	THD25279	CMLM*	
228.	Platyhelminthes	Trematoda	Digenea		Plagiorchiida	Troglotrematidae	***Paragonimus kellicotti***	txid100269	1	no	568	yes	KAF6777452	CMLM*	
229.	Platyhelminthes	Trematoda	Digenea		Strigeidida	Schistosomatidae	***Schistosoma japonicum***	txid6182	1	yes	586	yes	SKCS01000094 / GEZP01015410	CHLM*	
230.	Platyhelminthes	Trematoda	Digenea		Strigeidida	Schistosomatidae	***Schistosoma mansoni***	txid6183	1	yes	593	yes	CABG01000021 / JI394212	CHLM*	
231.	Rotifera	Acanthocephala			Echinorhynchida	Pomphorhynchidae	***Pomphorhynchus laevis***	txid141832	2	yes/yes	617/622	yes	WNNJ01000655 / GIBA01007009 / /WNNJ01002410 / GIBA01032868	no/no	VASRLFNLFQNN* / SRPMDPSEKNSI*
232.	Rotifera	Acanthocephala			Oligacanthorhynchida	Oligacanthorhynch	***Macracanthorhynchus hirudinaceus***	txid1032456	1	no	615	yes	Struck et al., 2014 (7_3748)	no	VTSKFFGGVFRSKN*
233.	Rotifera	Eurotatoria	Bdelloidea		Adinetida	Adinetidae	***Adineta ricciae***	txid249248	1	no	447	no	HE703448	no	SDGRVVAEKVVSVK*
234.	Rotifera	Eurotatoria	Bdelloidea		Adinetida	Adinetidae	***Adineta steineri***	txid433720	1	yes	554	yes	CAF0885156/CAJOBB010001691	no	VVTEKTVTVK*
235.	Rotifera	Eurotatoria	Bdelloidea		Adinetida	Adinetidae	***Adineta vaga***	txid104782	2x2	yes	552/545	yes	CAWI020041575 / CAWI020041273 / CAWI020041136 / CAWI020039987	no/no	TDGRVVAEKIVTLK*/SDGRVVAEK IVTVK*

: Stopp codon; (CaaX): indicates that alternatively spliced transcripts encoding lamins with alternative C-termini contain in addition the sequence information for a CaaX terminus within their 3' UTR.

	Phylum	Class	Subclass	Infraclass	Order	Family	*Species*	NCBI:txid	# of lamins	Gene	# aa	ORF com-plete	Accession	CaaX	alt. C-term
236.	Rotifera	Eurotatoria	Bdelloidea		Philodinida	Philodinidae	***Didymodactylos carnosus***	txid1234261	2	yes/yes	559/572	yes/yes	CAF0965054/CAF1018543 // CAF0965054/CAF1018543	no/no	GRIVAEKTTTVK* / GHIVAEKTTTVK*
237.	Rotifera	Eurotatoria	Bdelloidea				***Rotaria magnacalcarata***	txid392030	1	no	539	yes	GDRE01016112	no	SDGRVIVEKTVTVK*
238.	Rotifera	Eurotatoria	Bdelloidea				***Rotaria socialis***	txid392032	1	no	539	yes	GDRD01008530	no	SDGHVIAEKTVTVK*
239.	Rotifera	Eurotatoria	Bdelloidea				***Rotaria sordida***	txid392033	1	no	551	yes	GDRH01043061	no	ADGRIVAEKTITIK*
240.	Rotifera	Eurotatoria	Bdelloidea				***Rotaria tardigrada***	txid392034	1	no	551	yes	GDRK01063502	no	ADGRVVAEKTITIK*
241.	Rotifera	Eurotatoria	Monogonata		Ploima	Brachionidae	***Brachionus angularis***	txid396692	1	no	642	yes	GIWD01005582	no	VNKFFNLWKN*
242.	Rotifera	Eurotatoria	Monogonata		Ploima	Brachionidae	***Brachionus calyciflorus***	txid104777	1	no	587	yes	GACQ01000254	no	ILVDKDANVSFI*
243.	Rotifera	Eurotatoria	Monogonata		Ploima	Brachionidae	***Brachionus koreanus***	txid1199090	1	no	635	yes	GBXV02022239	no	.VQKFLGLWKSQP*
244.	Rotifera	Eurotatoria	Monogonata		Ploima	Brachionidae	***Brachionus manjavacas***	txid667381	1	no	632	yes	GFGK01003326	no	.TSAVQKFLGLWK*
245.	Rotifera	Eurotatoria	Monogonata		Ploima	Brachionidae	***Brachionus paranguensis***	txid2599771	1	no	633	yes	GIYQ01032002	no	AVQKFLGLWK*
246.	Rotifera	Eurotatoria	Monogonata		Ploima	Brachionidae	***Brachionus plicatilis***	txid10195	1		633	yes	GELX01001682	no	.SSAVQKFLGLWK*
247.	Rotifera	Eurotatoria	Monogonata		Ploima	Brachionidae	***Brachionus rotundiformis***	txid96890	1	yes	632	yes	GINZ01000221 / JAESIW010000137	no	KFLGLWKTG*
248.	Rotifera	Eurotatoria	Monogonata		Ploima	Proalidae	***Proales similis***	txid360698	1	yes	616	yes	Kim et al., 2021	no	SSRKFFAFWK*
249.	Rotifera	Pararotatoria			Seisonacea	Seisonidae	***Seison nebaliae***	txid104778	2	yes	601/601	yes/yes	Maurer et al., 2021 BMC Genomics	CSEE*	IVVQYSMNCSEE*/IIGQKLVRSRKLN*//VVVQYAMEYNTVVE*/IIGQKLVKSRRLN*
250.		Micrognathozoa			Limnognathida	Limnognathidae	***Limnognathia maerski***	txid195507	1	no	554	yes	Launer et al.,2019 MICR_Limn c72404_g1_i1	CSLM	

: Stopp codon; (CaaX): indicates that alternatively spliced transcripts encoding lamins with alternative C-termini contain in addition the sequence information for a CaaX terminus within their 3' UTR.
